# Genotyping-by-Sequencing-Based Genome-Wide Association Studies of Fusarium Wilt Resistance in Radishes (*Raphanus sativus* L.)

**DOI:** 10.3390/genes12060858

**Published:** 2021-06-03

**Authors:** O New Lee, Hyunjin Koo, Jae Woong Yu, Han Yong Park

**Affiliations:** 1 College of Life Sciences, Sejong University, Seoul 05006, Korea; onewlee@sejong.ac.kr; 2 Department of Agricultural Biotechnology and Research, Institute of Agriculture and Life Sciences, Seoul National University, Seoul 08826, Korea; 9hj1221@gmail.com; 3 C&K Genomics, Seoul 05836, Korea; crow3legs@naver.com

**Keywords:** GWAS, GBS, radish (*Raphanus sativus* L.), *Fusarium oxysporum*, Fusarium wilt

## Abstract

Fusarium wilt (FW) is a fungal disease that causes severe yield losses in radish production. The most effective method to control the FW is the development and use of resistant varieties in cultivation. The identification of marker loci linked to FW resistance are expected to facilitate the breeding of disease-resistant radishes. In the present study, we applied an integrated framework of genome-wide association studies (GWAS) using genotyping-by-sequencing (GBS) to identify FW resistance loci among a panel of 225 radish accessions, including 58 elite breeding lines. Phenotyping was conducted by manual inoculation of seedlings with the FW pathogen, and scoring for the disease index was conducted three weeks after inoculation during two constitutive years. The GWAS analysis identified 44 single nucleotide polymorphisms (SNPs) and twenty putative candidate genes that were significantly associated with FW resistance. In addition, a total of four QTLs were identified from F2 population derived from a FW resistant line and a susceptible line, one of which was co-located with the SNPs on chromosome 7, detected in GWAS study. These markers will be valuable for molecular breeding programs and marker-assisted selection to develop FW resistant varieties of *R. sativus*.

## 1. Introduction

Radishes (*R. sativus* L., 2*n* = 18) belong to the Brassicaceae family, and are an economically important crop that is globally utilized. The swollen taproot, young leaves, fresh sprouts, and immature siliques are eaten as vegetables, and are consumed raw, pickled, dried, and cooked [1]. All radish types are cultivated as animal fodder, and oil radishes are used as a source of seed oil. Radishes contain valuable nutrients and phytochemicals such as minerals, glucosinolates, and flavonoids [2,3]. Radish production is estimated at approximately seven million tons/year for global production, and it represents about 2% of total worldwide vegetable production [4]. Furthermore, radishes are an important vegetable in Asia, especially in China, Japan, and Korea, and the estimated radish production areas in the three countries are 1.2 million, 33,000, and 20,000 hectares, respectively [5,6].

The genus *Raphanus* originated in the coastal region of the Mediterranean [7]. Radishes were introduced to China along the Silk Road about 2000 years ago, and to Japan and Korea about 1300 years ago [5,7,8,9]. Most scholars believe that the cultivated radish (*R. sativus*) originated from wild radishes (*R. raphanistrum* L.), while others contend that *R. sativus* is possibly a progeny resulting from the hybridization of *R. maritimus* and *R. landra*, which are both subspecies of *R. raphanistrum* [7]. However, studies of chloroplast DNA variation in radishes indicated that *R. raphanistrum* is not the maternal ancestor of the cultivated radish, and East Asian wild radishes have played a role in the establishment of the East Asian cultivated radish [10]. Another wild *Raphanus* species, *R. sativus* var. raphanistroides Makino, was naturally grown in the coastal areas of China, Japan, and Korea [7,11]. High-depth resequencing analyses revealed that Asian cultivated radishes were closely related to Asian wild radishes, but they were distinct from European cultivated radishes [12]. Moreover, radish cultivars have been assigned to the following five different varieties based on morphological traits [7,11,13]: *R. sativus* var. hortensis Becker (East Asian big, long radish), var. niger Kerner (black Spanish radish), var. chinensis Gallizioli (Chinese oil radish), var. *sativus* L. (syn. var. *radicular* Pers.) (European small radish), and var. caudatus Hooker & Anderson (rat tail radish or feed radish).

Fusarium wilt (FW) of radish is a soil-borne disease caused by the fungal pathogen *Fusarium oxysporum* f. sp. *raphani*. FW causes severe yield losses in radish production in continuous cropping fields [14,15,16,17]. Disease symptoms include the yellowing and dropping of young leaves, vascular discoloration, and severe stunting, and infected plants eventually wilt and die as the disease progresses [18]. The FW pathogen can survive for long periods in the soil without a suitable host plant. Biotic and abiotic dispersal mechanisms and agronomic control were not sufficiently efficacious for disease prevention. Therefore, the preferred method for FW disease control is the development and use of FW-resistant radish cultivars.

Disease resistance in plants can be regulated by major genes, multiple additive genes, race-specific genes, or host-pathogen recognition genes [19]. FW resistance in radishes was reported to be controlled by quantitative genes with dominant or recessive interactions [5,20,21]. Genetic analysis using the inbred line raised from the Japanese ‘Risoh’ varietal group presented three complementary recessive resistance genes conferred FW resistance [3,21]. Molecular mapping has been successfully used to elucidate the FW resistance quantitative trait locus (QTL). The QTL for FW were detected in BC_1_S_1_ families and OPJ14 of the RAPD locus on LG1, which explained 60.4% of the phenotypic variation [5]. The results of the comparative mapping of this locus in *R. sativus* and *Arabidopsis thaliana* suggested that the locus corresponded to the long arm of *A. thaliana* chromosome 1, which contained *TIR-NBS* genes that are implicated in disease resistance [22]. QTL mapping using an RIL population resulted in the detection of two QTLs for FW resistance and one QTL for FW susceptibility, which were detected on LG1 and LG7, respectively [5]. Another QTL analysis using F2 populations led to the identification of eight QTLs for FW resistance, which were identified on LG 2, 3, 6, and 7 [17]. The results of a synteny analysis indicated that the QTL on LG 3 (with high logarithm of the odds (LOD) score) was homologous to chromosome 3 of *A. thaliana*, which contains disease-resistance gene clusters.

Despite several QTL studies that examined FW resistance, the associated genetic features in radishes are not completely understood. QTL mapping approaches using recombinant inbred lines (RIL) populations derived from biparents are based on limited allelic diversity and recombination [23]. Genome-wide association studies (GWAS) are powerful methods used to detect candidate markers related to complex traits, because the analyses screen a large number of individuals [24,25]. GWAS analyses have successfully identified the genetic basis of agronomic traits and the candidate genes of rice, maize, wheat, and soybeans [24,26]. In addition, validation of the mapped FW candidate markers identified from GWAS using another radish population is important to utilize those markers to radish breeding, because some of QTLs have not had consistent effects across environments. In this study, (1) a collection of 225 accessions was used to perform a GWAS analysis based on the genotyping-by-sequencing (GBS) approach to detect candidate markers to predict the loci associated with FW resistance in *R. sativus*, and (2) verify the effects of the FW markers using additional radish F2:3 population. These results may provide comprehensive insight into the genetic characteristics associated with FW resistance in *R. sativus*.

## 2. Materials and Methods

### 2.1. Plant Materials

The germplasm included a total of 225 *Raphanus* accessions that originated from 20 countries and included two species: *R. sativus* and *R. raphanistrum* (Supplementary Table S1). The majority of the accessions were *R. sativus* var. *sativus*, which accounted for 219 accessions. Moreover, *R. sativus* var. *albus*, *R. sativus* var. *caudatus*, *R. sativus* var. *niger*, and *R. raphanistrum* accounted for one, three, two, and two accessions, respectively.

A GBS-panel containing 225 radish accessions included 124 accessions provided by the National Agrobiodiversity Center (NAC)-RDA in Korea, 43 accessions provided by Genebank-NARO in Japan, and 58 elite breeding lines. The 124 accessions from NAC-RDA were selected from 818 accessions to maximize the phenotypic diversity (based on the genetic diversity of morphological characteristics), and plants were self-pollinated by single-seed descent for three generations. The 43 accessions from Genebank-NARO were selected from 419 accessions based on morphological characteristics such as root shape, leaf shape, and root skin color, and plants were self-pollinated by single-seed descent for one generation. In addition, 58 elite breeding lines that were used for commercial radish breeding programs were included, and plants were F8–10 progenies generated by single-seed descent.

Two radish inbred lines, G193 and G190, were selected from 225 GWAS accessions, and used to develop F2 population as parents. G193, the female parent, has a high level of resistance to Fusarium wilt. G190, the male parent, is susceptible to Fusarium wilt. Seeds from F1 plants from the cross of G193 and G190 were planted in the greenhouse at Sejong University. One hundred thirty-three random F2 seeds were planted in plastic pots and used for marker analysis and to produce F3 lines by single seed descent. F2 population has been used in genetic map construction by GBS. For QTL analysis, twenty-five seeds from each F3 lines were inoculated with fungal spores for evaluation of FW resistance in the greenhouse.

### 2.2. Pathogen Inoculation

**Figure 1:**
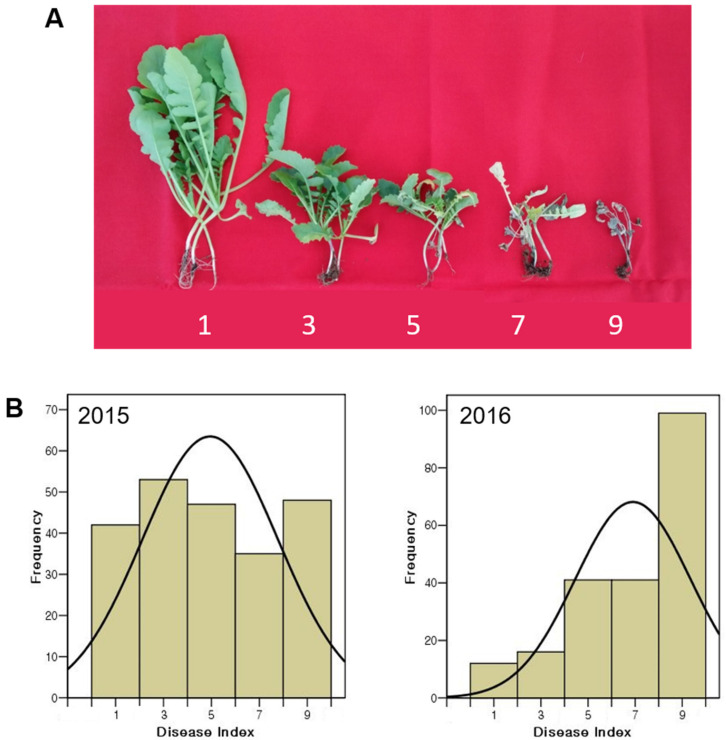
(**A**) Symptoms of Fusarium wilt of *R. sativus*. Plants were visually assessed for development of symptoms 2 and 3 weeks after inoculation with the pathogen. Symptom severity score was rated on a 5-point scale: 1 = healthy and no symptoms, 3 = chlorosis of lower leaves and slightly dwarfed, 5 = well developed symptoms of chlorosis, necrosis and dwarfed, 7 = severe symptoms of chlorosis, necrosis, and defoliation, 9 = plants died. (**B**) Frequency distributions of the Fusarium wilt disease in *R. sativus*. Disease index were evaluated in 2015 (left) and 2016 (right), respectively. The curve is the normal distribution line.

A panel of 225 radish accessions were evaluated for resistance to FW in September of 2015 and 2016 using the method described by Yu et al. (2013), with some modification [17]. Thirty seedlings from each accession were grown for seven days and inoculated with the FW pathogen using the root-cut dipping method [27]. Seven-day-old seedlings were lifted, and soil on the roots was removed with tapping fingers. The root-tips were cut to 2–3 cm in length, and were immediately immersed in a paper cup that contained 10 mL of Fusarium spore suspension solution, adjusted to a 10^6^ per mm^3^ concentration, for 10 min. Twenty-five seedlings were transplanted to plug trays containing commercial potting soil (Barokeo, Seoul Bio, Chungbuk, Korea). The temperature of the glasshouse was maintained between 26 °C and 29 °C, with natural light. Three weeks after inoculation, disease symptoms were scored on a disease index (DI) scale of 1, 3, 5, 7, and 9, and a DI value of 1 and 9 corresponded to the most resistant and most susceptible to FW, respectively (Figure 1A). The disease severity symptoms were follows: DI = 1, healthy and no symptoms; DI = 3, chlorosis of lower leaves and slightly dwarfed; DI = 5, well-developed chlorosis symptoms, necrosis, and dwarfed; DI = 7, severe symptoms of chlorosis, necrosis, and defoliation; DI = 9, plant death. F3 population was inoculated in September 2017 using the same method for 225 accessions.

### 2.3. Preparation of Genotyping-by-Sequencing (GBS) Libraries

Total genomic DNA was extracted from 0.1 g of leaf tissue using a DNeasy Plant Mini Kit (Qiagen, Hilden, Germany), following the manufacturer’s instruction. The amount of DNA was quantified using the standard procedure of the Quant-iT PicoGreen dsDNA Assay Kit (Molecular Probes, Eugene, OR, USA) with a Synergy HTX Multi-Mode Reader (Biotek, Winooski, VT, USA) that was normalized to 20 ng × μL^−1^. DNA (200 ng) was digested with 8 U of High-fidelity ApekI (New England BioLabs, Ipswich, MA, USA) at 75 °C for 2 h.

GBS DNA libraries were constructed according to previously described protocols [28,29], with minor modifications. The restriction digestion of DNA with ApekI was followed by ligation with adapters, which included a set of 96 different barcode-containing adapters used to tag individual samples and a common adapter for all samples. Ligation was performed using 200 cohesive end units of T4 DNA ligase (New England Biolabs, Ipswich, MA, USA) at 22 °C for 2 h, and the ligase was inactivated by incubation at 65 °C for 20 min. Sets containing 95 ligations were pooled into one sample and were then purified using a QIAquick PCR Purification Kit (Qiagen, Chatsworth, CA, USA). The pooled ligations (5 uL) were amplified in 50-uL reactions by multiplexing PCR using AccuPower Pfu PCR Premix (Bioneer, Daejeon, Korea) and 25 pmol of each primer. PCR cycles were as follows: 98 °C for 5 min; 18 cycles of 98 °C for 10 s, 65 °C for 5 s, and 72 °C for 5 s; and a final extension step at 72 °C for 5 min. PCR products were purified using a QIAquick PCR Purification Kit (Qiagen, Chatsworth, CA, USA), and were then evaluated with a BioAnalyzer 2100 (Agilent Technologies, Santa Clara, CA, USA) based on the distribution of fragment sizes. GBS libraries were sequenced with an Illumina NextSeq 500 (Illumina, San Diego, CA, USA), and 150-bp single-end reads were generated for 225 DNA samples for GWAS analysis, and 133 F2:3 DNA samples for QTL analysis.

### 2.4. Sequencing and Genotyping

Raw data was demultiplexed to sort samples using the GBSX tool [30], and chromosome pseudomolecule level genome data (Rs 1.0 Chromosome) from the Radish Genome Database (http://radish-genome.org; accessed on 27 July 2016) was used as a reference [31]. Following demultiplexing, single-end sequence reads were mapped to the radish reference genome using Bowtie2 [32], Genome Analysis Toolkit (GATK) and Picard Tools [33], which were used to locally realign reads in order to correct misalignments caused by insertions and deletions. GATK ‘RealignerTargetCreator’ and ‘IndelRealigner’ sequence data processing tools were used to call variants. Subsequently, GATK ‘HaplotypeCaller’ and ‘SelectVariants’ instructions were used to call variants. For QTL analysis, reads from two parents (G193, G190) were incorporated to call variants from local realignment of reads using GATK ‘RealignerTargetCreator’ and ‘IndelRealigner’. Variants were further filtered using GATK ‘FilterVariants’ instructions to filter out variants with quality score less than 30 (“QUAL < 30”), a Fisher score larger than 200 (“FS > 200.0”) and a quality depth less than 5 (“QD < 5.0”). An additional filtering step was conducted by VCFtools to remove SNPs with Minor Allele Frequency (MAF) less than 0.05 (“--maf 0.05”), ratio of missing genotype along samples larger than 0.05 (“--max-missing 0.95”), and average read depth less than 5 (“--min-meanDP 5”) [34].

### 2.5. Imputation and SNP Filtering

A total of 211,499 “raw” SNPs were identified after calling variants. Of these 211,499 SNPs, 188,813 SNPs were mapped to assembled chromosomes, and excluded markers (22,686 SNPs) mapped to scaffolds that were not assigned to a chromosome. SNPs with minor allele frequencies (MAF; <0.05) and call rates <80% were discarded using PLINK [35]. After filtering, missing genotypes were imputed using BEAGLE v4.1 with default settings [36].

### 2.6. Population Structure, Linkage Disequilibrium, and Genetic Diversity

STRUCTURE version 2.3.4 (http://pritchardlab.stanford.edu/structure.html; accessed on 29 August 2016) was used to determine population stratification patterns with a model-based approach [37]. Five runs were performed with 32,778 markers and k-values ranging from 2 to 14. To achieve a more accurate result, we used a 5000 burn-in period followed by a 50,000 MCMC (Markov Chain Monte Carlo) iteration parameter. The most likely subgroups were determined by measuring the estimated likelihood values (delta K). Hierarchical cluster analyses of 225 radish accessions were conducted using SNPRelate in the R package [38]. Linkage disequilibrium (LD) between marker loci on each chromosome was assessed with the squared allele frequency correlation using standalone TASSEL v.5.0 [39]. The genetic diversity index was used to indicate the level of differentiation in the population. Genetic population diversity was estimated using Nei’s gene diversity, which was calculated using Power Marker 3.25 [40,41]. SPSS 12.0KO for windows was used for statistical analyses (SPSS Inc., Chicago, IL, USA).

### 2.7. Genome-Wide Association Analysis

An association analysis was conducted in the standalone version of TASSEL v.5.0, and the kinship (K) matrix was estimated based on familial relatedness between lines in an identical-by-state (IBS) matrix. To solve the problem of skewed trait distribution, we conducted transformations using the box-cox function in the R package [42]. We then fit an MLM model with corrections for both population structure and relatedness (the QK model) [43]. Critical *p*-values (−log_10_ *p* ≥ 4) were used to detect significant Fusarium resistance markers [44]. The loci associated with significant SNPs were used to predict the candidate genes via annotation using the Radish Genome Database (http://radish-genome.org/Data_resource; accessed on 18 January 2017).

### 2.8. Linkage Map Construction & QTL Mapping

Custom code for transforming VCF formatted SNP data into input format for R/Qtl package [45]. Markers with duplicated pattern or distorted segregation ratios by Chi-square test with a *p*-value threshold of 0.05 were filtered out. R package “ASMap” was used to construct a linkage map with a *p*-value threshold of 1 × 10^−6^ and objective function as maximum likelihood with reordering of SNP markers according to their recombination frequency. The composite interval mapping (CIM) function in R/Qtl package was used for QTL mapping, with Kosambi function. QTLs inferred from the position of peaks with local maximum LOD values which exceeded 2.5 were selected to report Percentage of Variance Explained (PVE) and *p*-value tested with chi-square and F-statistics [46,47]. Confidence intervals of each QTL peaks were determined by “lodint” function in R/Qtl package, defined by LOD difference with its neighboring marker not to exceed 0.5.

## 3. Results

### 3.1. Phenotypic Variation

We evaluated Fusarium wilt (FW) resistance to *F. oxysporum* f. sp. *raphani* in 225 *R. sativus* accessions in 2015 and 2016. The disease index (DI) of Fusarium wilt was ranged from 1 to 9, with an average of 4.95 ± 2.83 in 2015 and with an average of 6.90 ± 2.45 in 2016 (Figure 1B). Phenotypic variations ranged from mid-resistant to susceptible. ANOVA of DI revealed significant differences among the genotypes (*F* = 2.615, *p* < 0.01), and between the two experimental years (*F* = 59.058, *p* < 0.01). Pearson’s correlation between the data in 2015 and 2016 showed 65.2%. FW resistance assessment in 225 accessions exhibited continuous variation in both years, suggesting that FW resistance was controlled by multiple genes. Ten accessions of 225 radish germplasms revealed strong resistance (DI = 1) to FW, whereas 39 accessions exhibited susceptibility (DI = 9) throughout two-year experiments.

### 3.2. Characterization and Distribution of Markers in the Radish Genome

Sequencing of the GBS library generated 362.5 million reads for the 225 lines comprised in three 96-plex libraries. About 77.8% of the reads were successfully aligned to the radish reference genome of Rs 1.0 Chromosome in radish genome database (http://radish-genome.org/Data_resource; accessed on 27 July 2016). The number of reads per individual distributed between 0.75 and 3.6 million (Supplementary Figure S1). Initially, a total of 211,499 “raw” SNPs were called from sequence reads, and 32,778 SNPs were identified after filtration.

### 3.3. Population Structure and Linkage Disequilibrium with Genotypic Data

**Figure 2:**
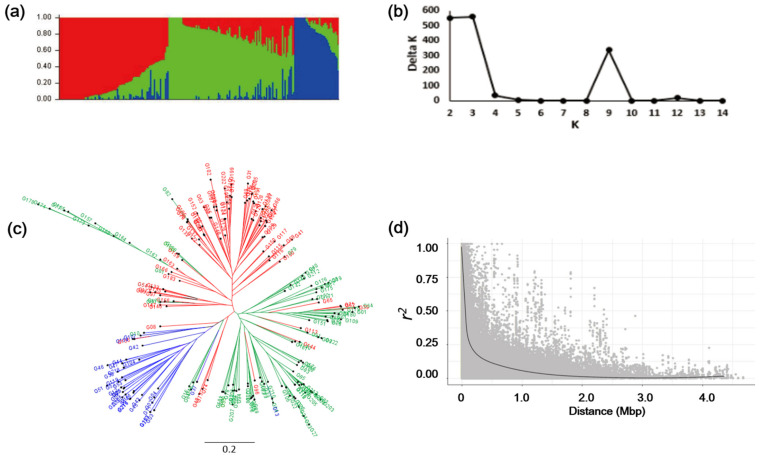
Genetic diversity and population structure.a (**a**) Population structure of 225 radish accessions. One cluster is represented by color (**b**) estimated delta K ranging from 2 to 14. (**c**) Neighbor-joining tree of all 225 radish accessions constructed by SNP markers. (**d**) Estimated linkage disequilibrium decay. Scatter plot showing the linkage disequilibrium (LD) decay across the chromosomes of radish.

A total of 32,778 markers were detected with the GBS approach after filtering options (Supplementary Figure S1). Population structure was assessed by calculating the ad hoc statistic (delta K) to identify the subpopulations within the 225 individuals. K values ranged from 2 to 14 on the entire dataset using filtered SNP markers. Based on changing delta K, estimated likelihood (LnP (D)) was found to be greatest when K = 3, suggesting that the population used in this study can be divided into three clusters (Figure 2a–c; Supplementary Figure S2). The cluster I, II, and III were admixtures. Each of the three clusters included different numbers of accessions: 89, 102, and 36 accessions, respectively. This information was utilized by in covariate Q and kinship matrices provided estimates of the relatedness among individuals. The 225 radish accessions were mainly divided into three clusters based on the geographical origins: (I) the south Chinese and Japanese radishes, (II) the north Chinese and Korean radishes, and (III) the European, Middle East radishes. *R. raphanistrum*, *R. sativus* var. albus and *R. sativus* var. niger were grouped together in cluster III, while *R. sativus* var. *caudatus* were grouped in cluster I. For controlling false positive problems, we used a population structure covariate in association analysis. LD decay was shown as the scatter plots of squared allele frequency correlation (*r*^2^) between the SNP loci in Figure 2d. Overall, in the genotypes, the distribution of *r*^2^ rapidly declined with increasing the physical distance. The LD varied all chromosomes decayed to <0.2 at about 226 kb (Figure 2d). In this study, Nei’s gene diversity and PIC values among 225 radish accessions were 0.321 and 0.260, respectively.

### 3.4. Genome-Wide Association Study (GWAS)

**Figure 3:**
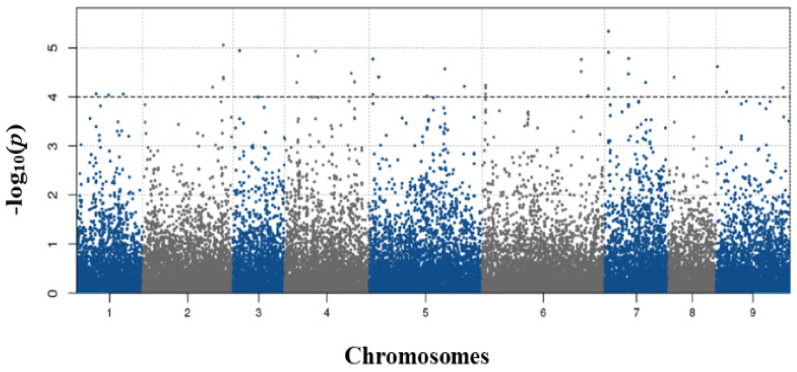
Manhattan plots of radish SNP markers mapped on nine chromosomes of *R. sativus* using mixed model (Q + K).

GWAS was performed using a mixed linear model (MLM) with corrections for population structure, relatedness among individuals, and year. GWAS identified 34 significant SNPs, which were matched to 20 candidate genes distributed on all chromosomes (Figure 3; Table 1). In addition, eight significant SNPs located in un-genetic regions were identified on chromosomes 1, 2, 4, 5, and 6 (Supplementary Table S2). Theses SNPs represent a minimum allele frequency (MAF) ranging from 0.05 and with a highest value of 4.6 × 10^−6^.

To search for the potential candidate genes related with disease resistance, we arbitrarily checked 30 kb and 225 kb upstream and downstream regions of the significant SNP loci located in the genic region, respectively (Supplementary Tables S3 and S4) [48]. We could detect 181 genes within 30 kb upstream and downstream region of SNPs located in the genic region (Supplementary Table S3). In addition, 40 genes associated with disease resistant proteins (TIR-NBS-LRR, F-box, and zinc finger) were identified among 1261 genes within 225 kb region of SNPs located in genic region (Supplementary Table S4).

### 3.5. QTL Mapping for Fusarium Wilt Resistance

To validate the significant markers detected from GWAS, we conducted QTL analysis associated with FW resistance. Firstly, we derived a 133 F2 population derived from ‘G193′ (resistant) and ‘G190′ (susceptible), and constructed a genetic map by genotyping by sequencing (GBS). From the 133 samples, 2 runs of sequencing produced about 359 million (~54.3 Gb) DNA reads. Among them, 8.56 million reads (~2.38%) failed to determine samples while demultiplexing steps. In average, 2.42 million reads per sample was used for mapping to the pseudomolecule reference sequence of radish, recording 79.0% of the overall alignment rate (215,271,639 reads mapped among 272,595,847 reads). The initial variant calling pipeline produced 261,769 variants including InDel, and continued filtering steps with GATK, finally reporting 118,407 SNPs. To reduce the number of SNPs for linkage mapping, we conducted further filtering steps with VCFtools to control MAF, missing rate, and read depth level, which resulted in 2372 SNPs. Finally, duplication and segregation ratio filtering resulted in 2248 SNPs for linkage map construction (Supplementary Figure S2). The length of resulted linkage maps spanned from 308 cM to 701 cM, and markers were evenly distributed to chromosomes (R1:296, R2:334, R3:177, R4:158, R5:426, R6:303, R7:145, R8:190, R9:164), and the resulting average distance between markers was to be 1.59~2.34 cM for all chromosomes.

Two parental lines showed contrasting characteristics for disease resistance (G193: DI 1, G190: DI 9). QTL analysis identified a total of 4 QTLs, conferring FW resistance that were distributed on chromosomes 3, 6, 7, and 8 (Table 2). The LOD values for four QTLs ranged from 2.58 to 6.70, and percentage of variance explained (PVE) ranged from 5.75% to 18.84%. *qFWR 3* on chromosome 7 explained most of the phenotypic variation of 18.84%, with an additive effect of 0.68, which co-located with the significant SNPs (GBS-FW 25 to 28) from GWAS study. These SNP markers were located in Rs404770, which encoded the glycosyltransferase family protein (*p* value = 6.85 × 10^−5^).

## 4. Discussion and Conclusions

Genome-wide association study has been used to detect putative functional markers and candidate genes related to complex traits of various plant species [25,49,50]. Genotyping-by-sequencing (GBS), high throughput and cost-effective genotyping methods, which can support GWAS even in species with limited genomic information [51]. In this study, we reported the results of GBS-GWAS in a panel of 225 radish accessions to identify single nucleotide polymorphism (SNP) and candidate genes associated with Fusarium wilt (FW) resistance.

In this study, a 225 GBS panel comprised of 167 accessions selected from 1237 radish germplasms based on their morphology, and 58 elite breeding lines, were evaluated for FW resistance (Supplementary Table S1). Ten accessions did not display FW symptoms throughout the 2-year experiments, which are originated from China, Korea, Thailand, Myanmar and Ghana. Meanwhile, 39 accessions were susceptible to FW in both years, which are originated Russia, Uzbekistan, Afghanistan, India, China, Korea and Japan. Disease severity of FW on the cultivars expressed the most difference in phenotypes of resistance and susceptibility to Fusarium wilt under 25 °C [52]. Dispersal of plant pathogens in soil is dependent on many environmental factors including temperature, relative humidity, pH level and soil particle size [53,54]. In 2015 and 2016, radish seedlings of 225 accessions were inoculated in the same way in a glasshouse maintained between 26 °C and 29 °C, with natural light. However, the outbreak of the disease was severe in 2016, which suggests that there may have been effects of environmental factors such as temperature and relative humidity on disease development and transmission.

Radishes have been cultivated around the world and diversified in terms of their morphology, i.e., root and leaf shape, root size and root color [55]. For example, European radish roots tend to be small, while East Asian radishes roots show diverse root size. The average genetic diversity among the four regional groups (Europe, Middle East, South Asia and East Asia) did not showed significant differences [55]. There was an obvious correlation between their geographical provenance and genetic relationships. In this study, the Nei’s gene diversity among 225 accessions was 0.321 (Figure 2c). It was similar to the genetic diversity of 0.358 for radish germplasms among worldwide accessions analyzed with AFLP markers. To compare the genetic diversity between the cultivars, it was higher than 0.296 for Europe, 0.267 for Middle Asia, 0.294 for South Asia and 0.297 for East Asia [55].

The GBS sequencing results were used to perform population structure analysis for the 225 accessions under an admixed model using the STRUTURE program. In the analysis of population structure, three clusters were confirmed (K = 3) (Figure 2a,b). Most of the accessions from North East Asian countries are grouped in cluster I and II, whereas the accessions from West Asian countries, Middle Eastern countries and European countries are mainly discriminated in cluster III. East Asian cultivated radishes were divided into two groups and were distinct from West Asian and European cultivated radishes. This is consistent with that Asian cultivated radishes are closely related to wild Asian radishes, but they are distinct from European/American cultivated radishes [12].

For linkage disequilibrium (LD), this was affected by many different factors, such as natural selection, domestication, founding events, genetic diversity, and population stratification [43,56,57]. In maize, the average decline of LD distance was 5–100 kb from the SNP analysis of genetic diversity using 447 germplasm [58,59]. Long LD decay was observed in self-pollinated crops in soybean (125–600 kb) and Arabidopsis (10–250 kb) [60]. LD decay is very rapid in out-crossing species and it is varied across the germplasms characteristics [61]. Usually, the crop had experienced less recombination, which contained more common alleles and long LD decay [58,59]. In this study, LD decay distance of radish for *r*^2^ greater than 0.2 was 226 kb, although it is an out-crossing crop (Figure 2d). The long LD decay could be related with the 58 breeding lines of 225 radish accessions, which were raised up by self-pollination over 10 times, and other accessions were also self-pollinated for once to three times to improve seed uniformity and propagation.

In this study, a GWAS identified 42 genomic regions strongly associated with FW resistance using the MLM (QKmodel) (Figure 3; Table 1). In multiple previous association analysis study, due to a leading false positive problem of GLM, MLM was thought to be more acceptable than the GLM method [62,63]. Rs498780 (GBS-FW33) on chromosome 9 encoded the TIR-NBS-LRR class disease resistance protein. It has been known to play an important role in controlling disease interacting or tolerance, having relations with development and growth functions, and in response to biotic and abiotic stress conditions [58,64]. In the result of Chinese cabbage, the TIR-NBS-LRR protein was suggested as a putative R gene for fusarium yellows resistance protein. From the additional analysis, they found that the candidate genes differentially expressed between FW susceptible and resistant lines [65].

In synteny analysis, the positions of the significant SNP markers for FW resistance were compared with the FW QTLs reported in previous studies by using marker information [17] (Supplementary Figure S3). The location of the FW QTLs on radish genomes were determined based on the sequence information of molecular markers used in a previous study. Yu et al. (2013) detected a total of eight loci conferring FW resistance, that were distributed on four LGs, namely, 2, 3, 6, and 7. Here, six SNPs located in genic and intergenic region were co-localized with the three FW QTLs (Supplementary Figure S3, Table 1 and Table S2). GBS-FW37 on chromosome 2 was co-located with *qFW6* on LG4 (Supplementary Figure S3a). One SNP on chromosome 5 and three SNPs on chromosome 6 were co-localized with *qFW4* (*Fwr1*) on LG3 (marker interval: ACMP0606-cnu_mBRPGN0085) which exhibited high LOD value ranging 4.34 to 8.84 during three trials in the previous study (Supplementary Figure S3c). In addition, GBS-FW33 on chromosome 9 was closely localized with *qFW7* on LG6 (Supplementary Figure S3d).

Recently, a major FW resistance QTL (*Fwr1*) on chromosome 5 was finely mapped [66]. A gene encoding a serine/arginine-rich protein kinase was suggested as a candidate gene for *Fwr1* by sequencing and expression analyses. Here, GBS-FW39 located in intergenic region was most close to *Fwr1* on chromosome 5 (Supplementary Figure S3). Following validation of SNPs using an F2 population, a total of four QTLs conferring FW resistance were detected on chromosomes 3, 6, 7, and 8, respectively (Table 2). Among them, *qFWR 3* on chromosome 7 (PVE 18.84%) was co-located with the significant SNPs (GBS-FW 25 to 28) from the GWAS study. These SNP markers were located in Rs404770, which gene encoded the glycosyltransferase family protein. Glycosyltransferase gene sequences were retrieved from NCBI databases for in silico-based screening of biotic stress-responsive genes [67]. In flax seeds, the overexpression of the glycosyltransferase gene resulted in a significant increase (73%) in the transgenic plant resistance to *F. oxysporum* infection [68].

We arbitrarily searched potential candidate genes in 225 kbp upstream and downstream of the 44 SNP loci (Supplementary Table S4). Total 1261 genes were identified, and 40 genes are considered to associate with disease resistance, such as LRR and zinc finger proteins. Leucine-rich repeat receptor-like kinase (LRR-RLK) plays a crucial role in plant disease resistance pathways [69]. Multiple LRR-RLKs were reported to control disease interactions and tolerance, having relations with development and growth functions and being associated with both biotic and abiotic stress conditions [58,64]. In addition, zinc finger protein is known to play a role by encoding a variety of NBS-LRR type of proteins. Especially, since it has a crucial role in host–pathogen interactions to combine zinc finger protein with NBS-LRR domain, these genes could be proposed to have a connection to the FW resistance in radishes [70].

In *A. thaliana*, *RFO1* (resistance to *fusarium oxysporum 1*, At1G79670.1) on chromosome 1 contributes resistance to *F. oxysporum* f. sp. *matthioli* and f. sp. *raphani*, which are causal agents of FW in *Arabidopsis* and radish, respectively [71]. *RFO1* encodes a member of the wall-associated kinase family of receptor-like kinases (RLKs), and contributes to immunity against *F. oxisporum* infection in the root vascular cylinder [71,72]. Comparative genome analysis revealed synteny between the *Raphanus* genome and *A. thaliana* on chromosome 1 [17]. However, we did not detect a common genomic region in the *Raphanus* genome that was homologous to *RFO1* in *A. thaliana*, which is consistent with the previous reports [17]. On the other hand, the *Foc-Bol* region of *Brassica oleracea* was designated to be involved in *qFW4* in the previous study, and this region is designated to chromosome 5 of *Raphanus* genome using flanking marker information [17,73]. GBS-FW39 was identified in intergenic region of chromosome 5, which is closely located with *qFW4* (Supplementary Figure S3). Further validation of SNPs may improve our understanding on how radish sensitivity to FW is controlled.

## Figures and Tables

**Table 1:** Potential candidate genes related with Fusarium wilt resistance detected by GWAS.

Gene	SNP	Chromosome	Encoding	*p*-Value
Rs014470	GBS-FW1	1	Brassinosteroid signaling positive regulator (BZR1) family protein	9.13 × 10^−5^
Rs045120	GBS-FW2 GBS-FW3 GBS-FW4 GBS-FW5 GBS-FW6	2	1-phosphatidylinositol-3-phosphate 5-kinases	8.80 × 10^−6^, 3.98 × 10^−5^, 4.25 × 10^−5^
Rs118240	GBS-FW7	3	β-galactosidase 8	1.13 × 10^−5^
Rs173640	GBS-FW8	4	myb domain protein 121	1.17 × 10^−5^
Rs204510	GBS-FW9	4	acyl-n-acyltransferase with ring five phd-type zinc finger domain-containing protein	3.30 × 10^−5^
Rs207880	GBS-FW10 GBS-FW11 GBS-FW12 GBS-FW13	4	transcription factor bhlh51	4.92 × 10^−5^
Rs160720	GBS-FW14	4	pentatricopeptide repeat-containing protein	1.46 × 10^−5^
Rs223390	GBS-FW15	5	neutral invertase	3.91 × 10^−5^
Rs267700	GBS-FW16	5	basic helix-loop-helix (bHLH) DNA-binding superfamily protein	2.68 × 10^−5^
Rs277800	GBS-FW17	5	unknown function	6.09 × 10^−5^
Rs220650	GBS-FW18 GBS-FW19	5	homeobox-leucine zipper protein hdg8	1.69 × 10^−5^, 8.91 × 10^−5^
Rs357270	GBS-FW20 GBS-FW21 GBS-FW22	6	unknown function	1.72 × 10^−5^, 3.05 × 10^−5^
Rs360300	GBS-FW23	6	P-loop containing nucleoside triphosphate hydrolases superfamily protein	9.48 × 10^−5^
Rs384720	GBS-FW24	7	subtilase family protein	5.07 × 10^−5^
Rs404770	GBS-FW25 GBS-FW26 GBS-FW27 GBS-FW28	7	glycosyltransferase family protein	4.60 × 10^−6^, 1.23 × 10^−5^, 6.85 × 10^−5^
Rs393590	GBS-FW29 GBS-FW30	7	Vacuolar sorting protein 9 (VPS9) domain	1.64 × 10^−5^, 3.42 × 10^−5^
Rs410280	GBS-FW31	8	ankyrin repeat family protein	3.96 × 10^−5^
Rs448450	GBS-FW32	9	major facilitator superfamily protein	2.41 × 10^−5^
Rs498780	GBS-FW33	9	tir-nbs-lrr class disease resistance protein	6.49 × 10^−5^
Rs454240	GBS-FW34	9	RNA polymerase II transcription elongation factor	7.91 × 10^−5^

**Table 2:** QTL peak loci (LOD > 2.5) from Composite Interval Mapping (CIM), with three different phenotyping trial data. Position on genetic map and confidence interval (CI. low, CI. high) is indicated for each QTL peaks, as well as LOD, Percentage of Variance Explained (PVE), *p* value by Chi-square test and F-test.

**Name**	**Chromosome**	**Pos** **(cM)**	**CI.** **Low**	**CI.** **High**	**LOD**	**PVE** **(%)**	**Additive**	**Dominance**	***p*** **-Value** **(Chi2)**	***p*** **-Value** **(F)**
*qFWR 1*	3	60.1	57.0	61.9	2.58	5.75	0.33	0.02	1.9 × 10^−3^	2.9 × 10^−3^
*qFWR 2*	6	264.0	254.8	274.1	5.35	11.93	0.44	0.10	4.1 × 10^−6^	9.6 × 10^−6^
*qFWR 3*	7	45.7	43.8	49.5	6.70	18.84	0.68	0.11	8.1 × 10^−9^	2.9 × 10^−8^
*qFWR 4*	8	340.5	336.9	346.3	3.48	7.90	0.36	0.09	2.1 × 10^−4^	3.8 × 10^−4^

## Data Availability

No new data were created or analyzed in this study. Data sharing is not applicable to this article.

## Supplementary Materials

The following are available online at https://www.mdpi.com/article/10.3390/genes12060858/s1, Figure S1: (a) Number of single nucleotide polymorphisms (SNPs) detected on each chromosome of *R. sativus* (b) SNP density (number of SNPs per Mbp) of *R. sativus*. Figure S2: Linkage map and SNP position from the GBS. Each linkage group is matched to the chromosomal pseudomolecule sequence, as indicated above the plot. Positions of SNP markers are indicated as black horizontal line along the linkage groups. Figure S3: Comparison of the GWAS results with those of previous QTL mapping reported in Yu et al. (2013). (a) One SNP (GBS-FW37) located in intergenic region of chromosome 2 were closely localized with *qFW6*; (b,c) one SNP (GBS-FW39) located in intergenic region on chromosome 5 and three SNPs (GBS-FW20, 21, 22) located in genic region of chromosome 6 were co-located with FW resistance QTL (*qFW4*); (d) one SNP (GBS-FW33) located in genic region of chromosome 9 were closely located with *qFW7*. Table S1: List of 225 radish accessions and cluster position of every accession. Table S2: Significant SNPs located in intergenic region with Fusarium wilt resistance detected by GWAS. Table S3: Potential candidate genes located in the 30 kb upstream and downstream region of the SNPs associated with Fusarium wilt of radish. Table S4: Disease-related genes identified 225 kb upstream and downstream of the SNP loci associated with Fusarium wilt of radish.
