# Supplementary material for: Genotyping-by-Sequencing-Based Genome-Wide Association Studies of Fusarium Wilt Resistance in Radishes (Raphanus sativus L.)
Source: Genes (Basel). 2021 Jun 3;12(6):858. doi: 10.3390/genes12060858 (PMC8228987; doi:10.3390/genes12060858)
Supplement: Supplementary file 1 [file genes-12-00858-s001.zip › genes-1199702-supplementary.pdf]

Supplemental Materials

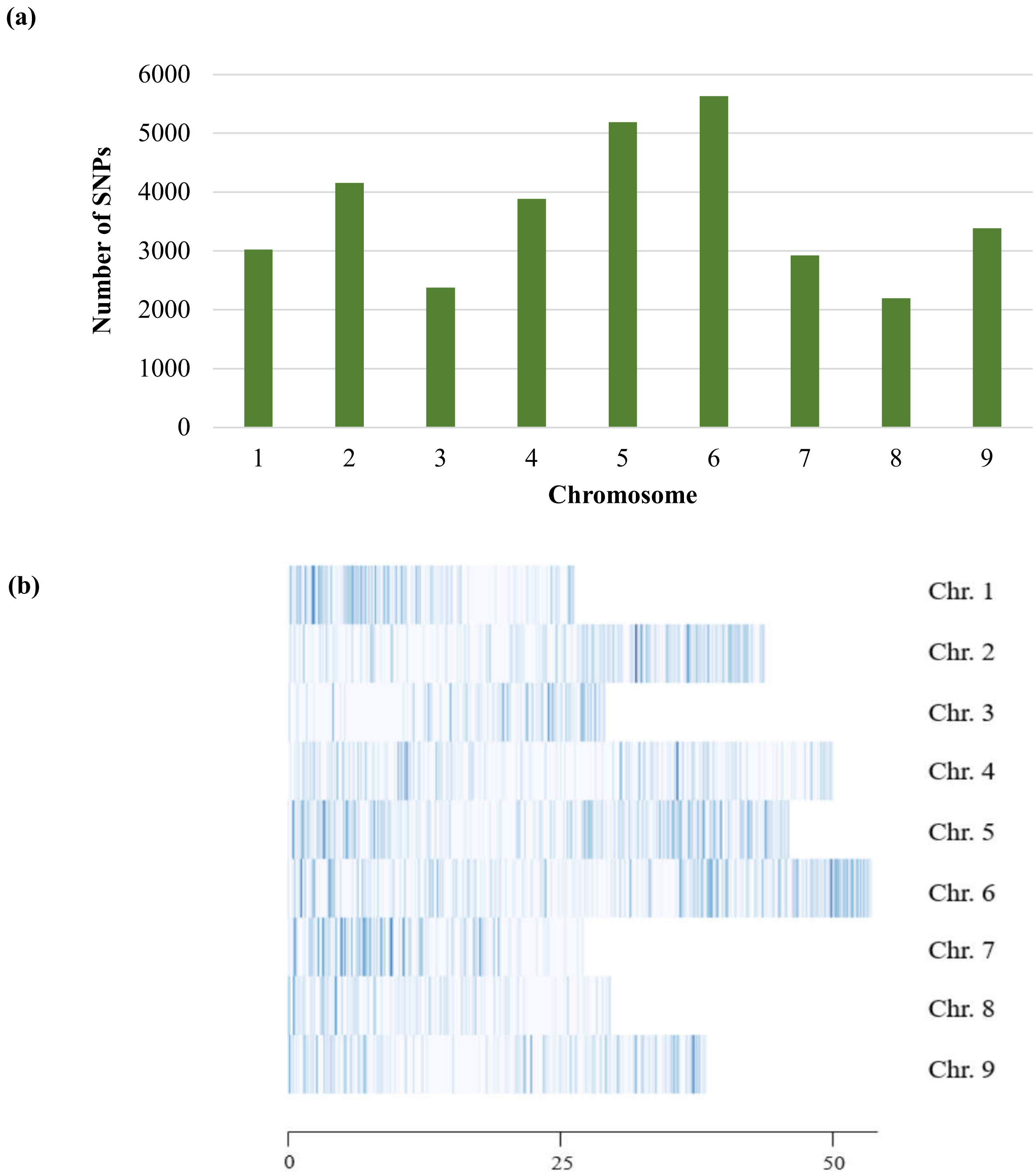

Figure S1. (**a**) Number of single nucleotide polymorphisms (SNPs) detected on each chromosome of *R. sativus* (**b**) SNP density (number of SNPs per Mbp) of *R. sativus*.

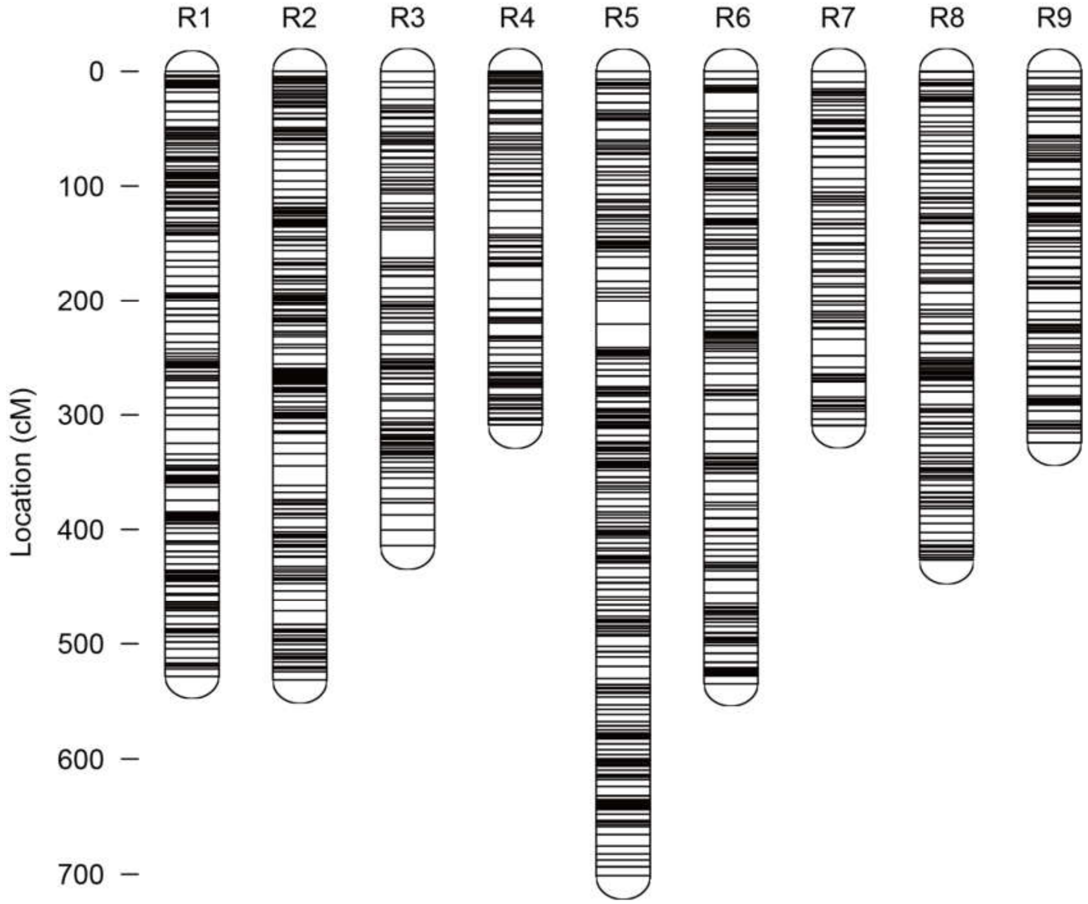

Figure S2. Linkage map and SNP position from the GBS. Each linkage group is matched to the chromosomal pseudomolecule sequence, as indicated above the plot. Positions of SNP markers are indicated as black horizontal line along the linkage groups.

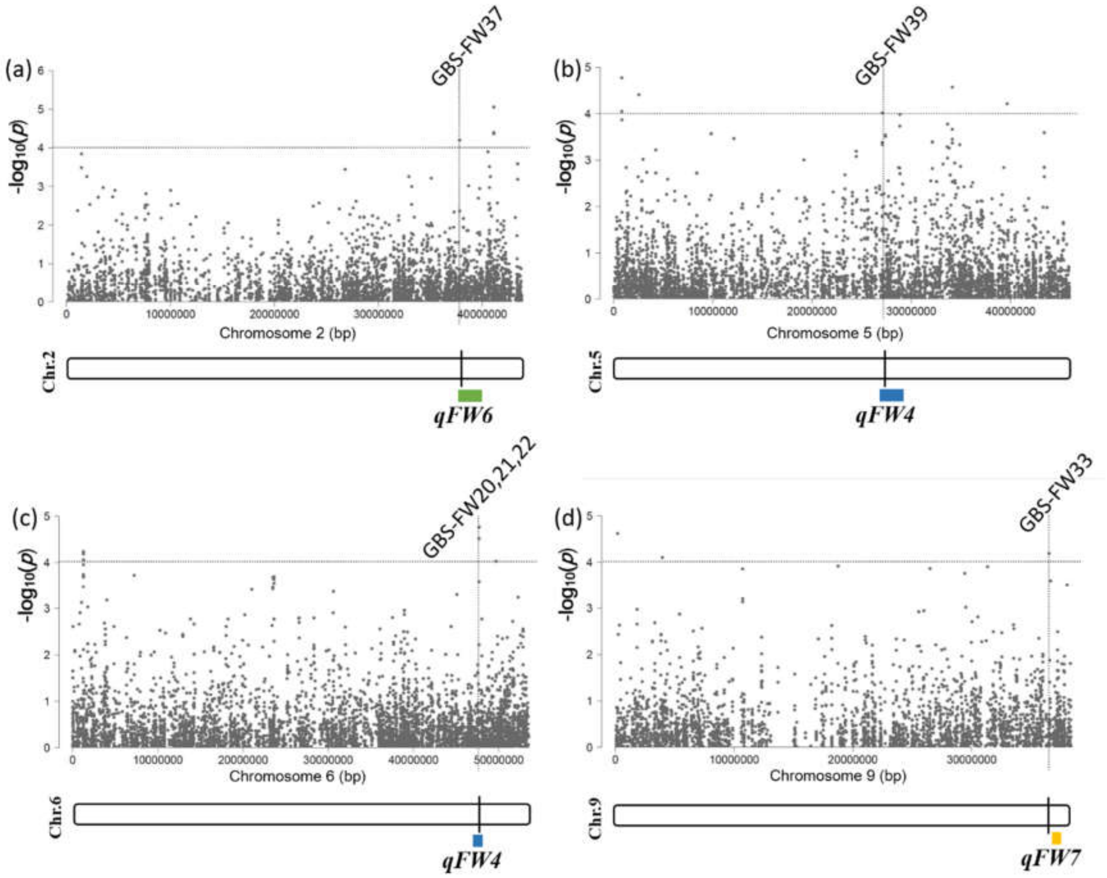

Figure S3. Comparison of the GWAS results with those of previous QTL mapping reported in Yu et al. (2013). (**a**) One SNP (GBS-FW37) located in intergenic region of chromosome 2 were closely localized with *qFW6*; (**b**,**c**) one SNP (GBS-FW39) located in intergenic region on chromosome 5 and three SNPs (GBS-FW20, 21, 22) located in genic region of chromosome 6 were co-located with FW resistance QTL (*qFW4*); (**d**) one SNP (GBS-FW33) located in genic region of chromosome 9 were closely located with *qFW7*.

Table S1. List of 225 radish accessions and cluster position of every accession.

Acc. No.	Scientific name	K = 3	ID	Origin	Source
G 1	Raphanus sativus var. sativus	2	IT 100598		NAC-RDA (Korea)
G 2	Raphanus sativus var. sativus	3	IT 102427	Turkey	NAC-RDA (Korea)
G 3	Raphanus sativus var. sativus	3	IT 102430	Turkey	NAC-RDA (Korea)
G 4	Raphanus sativus var. sativus	3	IT 102436	Turkey	NAC-RDA (Korea)
G 5	Raphanus sativus var. sativus	3	IT 102438	Turkey	NAC-RDA (Korea)
G 6	Raphanus sativus var. sativus	3	IT 102483	India	NAC-RDA (Korea)
G 7	Raphanus sativus var. sativus	3	IT 102483	India	NAC-RDA (Korea)
G 8	Raphanus sativus var. sativus	1	IT 102527	Russia	NAC-RDA (Korea)
G 9	Raphanus sativus var. sativus	2	IT 102554	China	NAC-RDA (Korea)
G 10	Raphanus sativus var. sativus	2	IT 203316	China	NAC-RDA (Korea)
G 11	Raphanus sativus var. sativus	3	IT 221487	Turkey	NAC-RDA (Korea)
G 12	Raphanus sativus var. sativus	3	IT 032530	Turkey	NAC-RDA (Korea)
G 13	Raphanus sativus var. sativus	2	IT 032541	India	NAC-RDA (Korea)
G 14	Raphanus sativus var. sativus	2	IT 032545	Turkey	NAC-RDA (Korea)
G 15	Raphanus sativus var. sativus	2	IT 203300	zbekistan	NAC-RDA (Korea)
G 16	Raphanus sativus var. sativus	2	IT 203310	yrgyzstan	NAC-RDA (Korea)
G 17	Raphanus sativus var. sativus	2	IT 210213	Russia	NAC-RDA (Korea)
G 18	Raphanus sativus var. sativus	2	IT 210213	Russia	NAC-RDA (Korea)
G 19	Raphanus sativus var. sativus	2	IT 210213	Russia	NAC-RDA (Korea)
G 20	Raphanus sativus var. sativus	2	IT 210213	Russia	NAC-RDA (Korea)
G 21	Raphanus sativus var. sativus	2	IT 210213	Russia	NAC-RDA (Korea)
G 22	Raphanus sativus var. sativus	2	IT 210213	Russia	NAC-RDA (Korea)
G 23	Raphanus sativus var. sativus	2	IT 210213	Russia	NAC-RDA (Korea)
G 24	Raphanus sativus var. sativus	2	IT 210213	Russia	NAC-RDA (Korea)

G 25	Raphanus sativus var. sativus	2	IT 210213	Russia	NAC-RDA (Korea)
G 26	Raphanus sativus var. sativus	2	IT 210213	Russia	NAC-RDA (Korea)
G 27	Raphanus sativus var. sativus	2	IT 213154	China	NAC-RDA (Korea)
G 28	Raphanus sativus var. sativus	2	IT 136486	Nepal	NAC-RDA (Korea)
G 29	Raphanus sativus var. sativus	2	IT 219316	Myanmar	NAC-RDA (Korea)
G 30	Raphanus sativus var. sativus	2	IT 208395	Nepal	NAC-RDA (Korea)
G 31	Raphanus sativus var. sativus	1	IT 250765	India	NAC-RDA (Korea)
G 32	Raphanus sativus var. sativus	2	IT 221952	China	NAC-RDA (Korea)
G 33	Raphanus sativus var. sativus	2	IT 247865	China	NAC-RDA (Korea)
G 34	Raphanus sativus var. sativus	2	IT 209112	Ghana	NAC-RDA (Korea)
G 35	Raphanus sativus var. sativus	1	IT 210211	Japan	NAC-RDA (Korea)
G 36	Raphanus sativus var. sativus	1	IT 250750	India	NAC-RDA (Korea)
G 37	Raphanus sativus var. sativus	3	IT 203313	zbekistan	NAC-RDA (Korea)
G 38	Raphanus sativus var. sativus	3	IT 032575	fghanistan	NAC-RDA (Korea)
G 39	Raphanus sativus var. sativus	2	IT 221953	China	NAC-RDA (Korea)
G 40	Raphanus sativus var. sativus	2	IT 250741	India	NAC-RDA (Korea)
G 41	Raphanus sativus var. sativus	1	IT 32549	India	NAC-RDA (Korea)
G 42	Raphanus sativus var. sativus	3	IT 032571	India	NAC-RDA (Korea)
G 43	Raphanus sativus var. sativus	3	IT 102451	Turkey	NAC-RDA (Korea)
G 44	Raphanus sativus var. sativus	3	IT 102479	Turkey	NAC-RDA (Korea)
G 45	Raphanus sativus var. sativus	3	IT 032672	Turkey	NAC-RDA (Korea)
G 46	Raphanus sativus var. sativus	3	IT 102502	Pakistan	NAC-RDA (Korea)
G 47	Raphanus sativus var. sativus	3	IT 032704	Iran	NAC-RDA (Korea)
G 48	Raphanus sativus var. sativus	2	IT 102523	Russia	NAC-RDA (Korea)
G 49	Raphanus sativus var. sativus	1	IT 200380	Nepal	NAC-RDA (Korea)
G 50	Raphanus sativus var. sativus	2	IT 250753	India	NAC-RDA (Korea)

G 51	Raphanus sativus var. niger	3	IT 218921	Italy	NAC-RDA (Korea)
G 52	Raphanus sativus var. sativus	3	IT 032714	Russia	NAC-RDA (Korea)
G 53	Raphanus sativus var. niger	3	IT 218920	Germany	NAC-RDA (Korea)
G 54	Raphanus sativus var. albus	1	IT 218927	Japan	NAC-RDA (Korea)
G 55	Raphanus sativus var. sativus	2	IT 250766	Italy	NAC-RDA (Korea)
G 56	Raphanus sativus var. sativus	2	IT 250785	Japan	NAC-RDA (Korea)
G 57	Raphanus sativus var. sativus	2	IT 250792	China	NAC-RDA (Korea)
G 58	Raphanus sativus var. sativus	1	IT 100638		NAC-RDA (Korea)
G 59	Raphanus sativus var. sativus	1	IT 100596		NAC-RDA (Korea)
G 60	Raphanus sativus var. sativus	2	IT 032708	Thailand	NAC-RDA (Korea)
G 61	Raphanus sativus var. sativus	2	IT 100586		NAC-RDA (Korea)
G 62	Raphanus sativus var. sativus	2	IT 136484	Nepal	NAC-RDA (Korea)
G 63	Raphanus sativus var. sativus	1	IT 136484	Nepal	NAC-RDA (Korea)
G 64	Raphanus sativus var. sativus	2	IT 221965	China	NAC-RDA (Korea)
G 65	Raphanus sativus var. sativus	1	IT 166997	China	NAC-RDA (Korea)
G 66	Raphanus sativus var. sativus	1	IT 100709		NAC-RDA (Korea)
G 67	Raphanus sativus var. sativus	1	IT 210223		NAC-RDA (Korea)
G 68	Raphanus sativus var. sativus	1	IT 100604		NAC-RDA (Korea)
G 69	Raphanus sativus var. sativus	1	IT 250773	India	NAC-RDA (Korea)
G 70	Raphanus sativus var. sativus	1	IT 100666		NAC-RDA (Korea)
G 71	Raphanus sativus var. sativus	2	IT 210057	Thailand	NAC-RDA (Korea)
G 72	Raphanus sativus var. sativus	1	IT 200369	Nepal	NAC-RDA (Korea)
G 73	Raphanus sativus var. sativus	1	IT 200370	Nepal	NAC-RDA (Korea)
G 74	Raphanus sativus var. sativus	1	IT 200370	Nepal	NAC-RDA (Korea)
G 75	Raphanus sativus var. sativus	1	IT 200371	Nepal	NAC-RDA (Korea)
G 76	Raphanus sativus var. sativus	1	IT 206774	Nepal	NAC-RDA (Korea)

G 77	Raphanus sativus var. sativus	2	IT 210237		NAC-RDA (Korea)
G 78	Raphanus sativus var. sativus	2	IT 102375	orea, South	NAC-RDA (Korea)
G 79	Raphanus sativus var. sativus	2	IT 191107	orea, South	NAC-RDA (Korea)
G 80	Raphanus sativus var. sativus	2	IT 100648		NAC-RDA (Korea)
G 81	Raphanus sativus var. sativus	2	IT 100632		NAC-RDA (Korea)
G 82	Raphanus sativus var. sativus	2	IT 100716		NAC-RDA (Korea)
G 83	Raphanus sativus var. sativus	2	IT 188102	China	NAC-RDA (Korea)
G 84	Raphanus sativus var. sativus	2	IT 220675	zbekistan	NAC-RDA (Korea)
G 85	Raphanus sativus var. sativus	2	IT 100715	orea, South	NAC-RDA (Korea)
G 86	Raphanus sativus var. sativus	2	IT 188101	China	NAC-RDA (Korea)
G 87	Raphanus sativus var. sativus	2	IT 188101	China	NAC-RDA (Korea)
G 88	Raphanus sativus var. sativus	2	IT 188101	China	NAC-RDA (Korea)
G 89	Raphanus sativus var. sativus	2	IT 171340	China	NAC-RDA (Korea)
G 90	Raphanus sativus var. sativus	1	IT 100634		NAC-RDA (Korea)
G 91	Raphanus sativus var. sativus	2	IT 112658	orea, South	NAC-RDA (Korea)
G 92	Raphanus sativus var. sativus	2	IT 210215	orea, South	NAC-RDA (Korea)
G 93	Raphanus sativus var. sativus	2	IT 100701	orea, South	NAC-RDA (Korea)
G 94	Raphanus sativus var. sativus	2	IT 100589	orea, South	NAC-RDA (Korea)
G 95	Raphanus sativus var. sativus	2	IT 100588	orea, South	NAC-RDA (Korea)
G 96	Raphanus sativus var. sativus	2	IT 102372	orea, South	NAC-RDA (Korea)
G 97	Raphanus sativus var. sativus	1	IT 215008	orea, South	NAC-RDA (Korea)
G 98	Raphanus sativus var. sativus	1	IT 215020	orea, South	NAC-RDA (Korea)
G 99	Raphanus sativus var. sativus	1	IT 215036	orea, South	NAC-RDA (Korea)
G 100	Raphanus sativus var. sativus	1	IT 100643	orea, South	NAC-RDA (Korea)
G 101	Raphanus sativus var. sativus	2	IT 101247	orea, South	NAC-RDA (Korea)
G 102	Raphanus sativus var. sativus	1	IT 210224		NAC-RDA (Korea)

G 103	Raphanus sativus var. sativus	3	IT 032553	Turkey	NAC-RDA (Korea)
G 104	Raphanus sativus var. sativus	3	IT 032558	Turkey	NAC-RDA (Korea)
G 105	Raphanus sativus var. sativus	3	IT 032606	Iran	NAC-RDA (Korea)
G 106	Raphanus sativus var. sativus	2	IT 213154	China	NAC-RDA (Korea)
G 107	Raphanus sativus var. sativus	3	IT 119240	Iran	NAC-RDA (Korea)
G 108	Raphanus sativus var. sativus	2	IT 209936	zbekistan	NAC-RDA (Korea)
G 109	Raphanus sativus var. sativus	2	IT 221954	China	NAC-RDA (Korea)
G 110	Raphanus sativus var. sativus	2	IT 100715	rea, South	NAC-RDA (Korea)
G 111	Raphanus sativus L. Daikon Group (= var. longipinnatus L. H. Bailey)	3	27292	USA	S-Genebank (Japan)
G 112	Raphanus sativus L. Daikon Group (= var. longipinnatus L. H. Bailey)	2	90768	China	S-Genebank (Japan)
G 113	Raphanus sativus L. Daikon Group (= var. longipinnatus L. H. Bailey)	1	27024	Japan	S-Genebank (Japan)
G 114	Raphanus sativus L. Daikon Group (= var. longipinnatus L. H. Bailey)	1	27138	Japan	S-Genebank (Japan)
G 115	Raphanus sativus L. Daikon Group (= var. longipinnatus L. H. Bailey)	1	27046	Japan	S-Genebank (Japan)
G 116	Raphanus sativus L. Daikon Group (= var. longipinnatus L. H. Bailey)	1	27184	Japan	S-Genebank (Japan)
G 117	Raphanus sativus L. Daikon Group (= var. longipinnatus L. H. Bailey)	1	27201	Japan	S-Genebank (Japan)
G 118	Raphanus sativus L. Daikon Group (= var. longipinnatus L. H. Bailey)	1	27047	Japan	S-Genebank (Japan)
G 119	Raphanus sativus L. Daikon Group (= var. longipinnatus L. H. Bailey)	3	27287	France	S-Genebank (Japan)
G 120	Raphanus sativus L. Daikon Group (= var. longipinnatus L. H. Bailey)	1	27039	Japan	S-Genebank (Japan)
G 121	Raphanus sativus L. Daikon Group (= var. longipinnatus L. H. Bailey)	1	27048	Japan	S-Genebank (Japan)
G 122	Raphanus sativus L. Daikon Group (= var. longipinnatus L. H. Bailey)	1	27154	Japan	S-Genebank (Japan)
G 123	Raphanus sativus L. Daikon Group (= var. longipinnatus L. H. Bailey)	1	26973	Japan	S-Genebank (Japan)
G 124	Raphanus sativus L. Daikon Group (= var. longipinnatus L. H. Bailey)	1	26996	Japan	S-Genebank (Japan)
G 125	Raphanus sativus L. Daikon Group (= var. longipinnatus L. H. Bailey)	1	26956	Japan	S-Genebank (Japan)
G 126	Raphanus sativus L. Daikon Group (= var. longipinnatus L. H. Bailey)	1	26957	Japan	S-Genebank (Japan)
G 127	Raphanus sativus L. Daikon Group (= var. longipinnatus L. H. Bailey)	1	27215	Japan	S-Genebank (Japan)
G 128	Raphanus sativus L. Daikon Group (= var. longipinnatus L. H. Bailey)	1	37663	Japan	S-Genebank (Japan)

G 129	Raphanus sativus L. Daikon Group (= var. longipinnatus L. H. Bailey)	1	27145	Japan	\S-Genebank (Japan)
G 130	Raphanus sativus L. Daikon Group (= var. longipinnatus L. H. Bailey)	1	26993	Japan	\S-Genebank (Japan)
G 131	Raphanus sativus L. Daikon Group (= var. longipinnatus L. H. Bailey)	1	26941	Japan	\S-Genebank (Japan)
G 132	Raphanus sativus L. Daikon Group (= var. longipinnatus L. H. Bailey)	1	27051	Japan	\S-Genebank (Japan)
G 133	Raphanus sativus L. Daikon Group (= var. longipinnatus L. H. Bailey)	1	27244	Japan	\S-Genebank (Japan)
G 134	Raphanus sativus L. Daikon Group (= var. longipinnatus L. H. Bailey)	1	26969	Japan	\S-Genebank (Japan)
G 135	Raphanus sativus L. Daikon Group (= var. longipinnatus L. H. Bailey)	1	27229	Japan	\S-Genebank (Japan)
G 136	Raphanus sativus L. Daikon Group (= var. longipinnatus L. H. Bailey)	1	27050	Japan	\S-Genebank (Japan)
G 137	Raphanus sativus L. Daikon Group (= var. longipinnatus L. H. Bailey)	1	27081	Japan	\S-Genebank (Japan)
G 138	Raphanus sativus L. Daikon Group (= var. longipinnatus L. H. Bailey)	1	26949	Japan	\S-Genebank (Japan)
G 139	Raphanus sativus L. Daikon Group (= var. longipinnatus L. H. Bailey)	1	27166	Japan	\S-Genebank (Japan)
G 140	Raphanus sativus L. Daikon Group (= var. longipinnatus L. H. Bailey)	2	27262	China	\S-Genebank (Japan)
G 141	Raphanus sativus L. Daikon Group (= var. longipinnatus L. H. Bailey)	3	73393	Egypt	\S-Genebank (Japan)
G 142	Raphanus sativus L. Daikon Group (= var. longipinnatus L. H. Bailey)	2	76701	China	\S-Genebank (Japan)
G 143	Raphanus sativus L. Daikon Group (= var. longipinnatus L. H. Bailey)	2	43244	China	\S-Genebank (Japan)
G 144	Raphanus sativus L. Daikon Group (= var. longipinnatus L. H. Bailey)	1	27219	Japan	\S-Genebank (Japan)
G 145	Raphanus sativus L. Daikon Group (= var. longipinnatus L. H. Bailey)	2	27263	China	\S-Genebank (Japan)
G 146	Raphanus sativus L. Daikon Group (= var. longipinnatus L. H. Bailey)	1	27254	Japan	\S-Genebank (Japan)
G 147	Raphanus sativus L. Daikon Group (= var. longipinnatus L. H. Bailey)	1	26947	Japan	\S-Genebank (Japan)
G 148	Raphanus sativus L. Daikon Group (= var. longipinnatus L. H. Bailey)	1	27159	Japan	\S-Genebank (Japan)
G 149	Raphanus sativus L. Daikon Group (= var. longipinnatus L. H. Bailey)	2	76700	China	\S-Genebank (Japan)
G 150	Raphanus sativus L. Daikon Group (= var. longipinnatus L. H. Bailey)	1	27071	Japan	\S-Genebank (Japan)
G 151	Raphanus sativus L. Daikon Group (= var. longipinnatus L. H. Bailey)	2	27277	Taiwan	\S-Genebank (Japan)
G 152	Raphanus sativus L. Daikon Group (= var. longipinnatus L. H. Bailey)	1	27200	Japan	\S-Genebank (Japan)
G 153	Raphanus sativus L. Daikon Group (= var. longipinnatus L. H. Bailey)	2	27265	China	\S-Genebank (Japan)
G 154	Raphanus sativus var. caudatus	3	SJ 1		Breeding lines
G 155	Raphanus sativus var. caudatus	1	SJ 2		Breeding lines

G 156	Raphanus sativus var. caudatus	1	SJ 3		Breeding lines
G 157	Raphanus sativus var. sativus	2	SJ 4		Breeding lines
G 158	Raphanus sativus var. sativus	2	IT 100673		NAC-RDA (Korea)
G 159	Raphanus sativus var. sativus	1	IT 210245		NAC-RDA (Korea)
G 160	Raphanus sativus var. sativus	2	IT 110841	orea, South	NAC-RDA (Korea)
G 161	Raphanus sativus var. sativus	2	IT 100672		NAC-RDA (Korea)
G 162	Raphanus sativus var. sativus	2	IT 100637		NAC-RDA (Korea)
G 163	Raphanus sativus var. sativus	1	SJ 5		Breeding lines
G 164	Raphanus sativus var. sativus	2	SJ 6		Breeding lines
G 165	Raphanus sativus var. sativus	2	SJ 7		Breeding lines
G 166	Raphanus sativus var. sativus	1	SJ 8		Breeding lines
G 167	Raphanus sativus var. sativus	1	SJ 9		Breeding lines
G 168	Raphanus sativus var. sativus	2	SJ 10		Breeding lines
G 169	Raphanus sativus var. sativus	2	SJ 11		Breeding lines
G 170	Raphanus sativus var. sativus	2	SJ 12		Breeding lines
G 171	Raphanus sativus var. sativus	2	SJ 13		Breeding lines
G 172	Raphanus sativus var. sativus	2	SJ 14		Breeding lines
G 173	Raphanus sativus var. sativus	2	SJ 15		Breeding lines
G 174	Raphanus sativus var. sativus	2	SJ 16		Breeding lines
G 175	Raphanus sativus var. sativus	2	SJ 17		Breeding lines
G 176	Raphanus sativus var. sativus	2	SJ 18		Breeding lines
G 177	Raphanus sativus var. sativus	1	SJ 19		Breeding lines
G 178	Raphanus sativus var. sativus	2	SJ 20		Breeding lines
G 179	Raphanus sativus var. sativus	2	SJ 21		Breeding lines
G 180	Raphanus sativus var. sativus	2	SJ 22		Breeding lines
G 181	Raphanus sativus var. sativus	2	SJ 23		Breeding lines
G 182	Raphanus sativus var. sativus	1	SJ 24		Breeding lines
G 183	Raphanus sativus var. sativus	1	SJ 25		Breeding lines
G 184	Raphanus sativus var. sativus	1	SJ 26		Breeding lines
G 185	Raphanus sativus var. sativus	1	SJ 27		Breeding lines
G 186	Raphanus sativus var. sativus	1	SJ 28		Breeding lines
G 187	Raphanus sativus var. sativus	1	SJ 29		Breeding lines
G 188	Raphanus sativus var. sativus	1	SJ 30		Breeding lines
G 189	Raphanus sativus var. sativus	1	SJ 31		Breeding lines
G 190	Raphanus sativus var. sativus	1	SJ 32		Breeding lines
G 191	Raphanus sativus var. sativus	1	SJ 33		Breeding lines
G 192	Raphanus sativus var. sativus	1	SJ 34		Breeding lines
G 193	Raphanus sativus var. sativus	1	SJ 35		Breeding lines
G 194	Raphanus sativus var. sativus	1	SJ 36		Breeding lines
G 195	Raphanus sativus var. sativus	1	SJ 37		Breeding lines
G 196	Raphanus sativus var. sativus	1	SJ 38		Breeding lines
G 197	Raphanus sativus var. sativus	1	SJ 39		Breeding lines
G 198	Raphanus sativus var. sativus	1	SJ 40		Breeding lines
G 199	Raphanus sativus var. sativus	1	SJ 41		Breeding lines
G 200	Raphanus sativus var. sativus	1	SJ 42		Breeding lines
G 201	Raphanus sativus var. sativus	1	SJ 43		Breeding lines

G 202	Raphanus sativus var. sativus	1	SJ 44		Breeding lines
G 203	Raphanus sativus var. sativus	2	SJ 45		Breeding lines
G 204	Raphanus sativus var. sativus	2	SJ 46		Breeding lines
G 205	Raphanus sativus var. sativus	2	SJ 47		Breeding lines
G 206	Raphanus sativus var. sativus	2	SJ 48		Breeding lines
G 207	Raphanus sativus var. sativus	2	SJ 49		Breeding lines
G 208	Raphanus sativus var. sativus	2	SJ 50		Breeding lines
G 209	Raphanus sativus var. sativus	1	SJ 51		Breeding lines
G 210	Raphanus sativus var. sativus	2	SJ 52		Breeding lines
G 211	Raphanus sativus var. sativus	2	SJ 53		Breeding lines
G 212	Raphanus sativus var. sativus	2	SJ 54		Breeding lines
G 213	Raphanus sativus var. sativus	2	SJ 55		Breeding lines
G 214	Raphanus sativus var. sativus	3	SJ 56		Breeding lines
G 215	Raphanus sativus var. sativus	3	SJ 57		Breeding lines
G 216	Raphanus sativus var. sativus	3	SJ 58		Breeding lines
G 217	Raphanus sativus var. sativus	3	IT 032553	Turkey	NAC-RDA (Korea)
G 218	Raphanus sativus var. sativus	3	IT 032578	fghanistan	NAC-RDA (Korea)
G 219	Raphanus sativus var. sativus	2	IT 100715	orea, South	NAC-RDA (Korea)
G 220	Raphanus sativus var. sativus	2	IT 209936	zbekistan	NAC-RDA (Korea)
G 221	Raphanus raphanistrum subsp. raphanistrum	3	IT 218913	France	NAC-RDA (Korea)
G 222	Raphanus sativus var. sativus	2	IT 102554	China	NAC-RDA (Korea)
G 223	Raphanus sativus var. sativus	3	IT 032606	Iran	NAC-RDA (Korea)
G 224	Raphanus raphanistrum subsp. landra	3	IT 218912	Italy	NAC-RDA (Korea)
G 225	Raphanus sativus var. sativus	1	IT 206774	Nepal	NAC-RDA (Korea)

Table S2. Significant SNPs located in intergenic region with Fusarium wilt resistance detected by GWAS.

SNP	Chromosome	*p*-Value
**GBS-FW35**	1	8.63E-05
**GBS-FW36**	1	8.71E-05
**GBS-FW37**	2	6.34E-05
**GBS-FW38**	4	5.07E-05
**GBS-FW39**	5	9.67E-05
**GBS-FW40**	6	5.80E-05
**GBS-FW41**	6	6.58E-05
**GBS-FW42**	6	8.72E-05

Table S3. Potential candidate genes located in the 30kb upstream and downstream region of the SNPs associated with Fusarium wilt of radish.

No.	GeneID	SNP	Function
**1**	Rs014430	R1_8001479	wall-associated receptor kinase-like 11 [BLAST2GO] ǀ Wall-associated kinase family protein [AT1G19390]
**2**	Rs014520	R1_8001479	thaumatin-like protein [BLAST2GO] ǀ Best-Hit: Pathogenesis-related thaumatin superfamily protein [AT1G19320]
**3**	Rs014440	R1_8001479	Unknown protein
**4**	Rs014510	R1_8001479	Unknown protein
**5**	Rs014450	R1_8001479	uncharacterized membrane protein at1g75140-like [BLAST2GO] ǀ Best-Hit: unknown protein [AT1G19370]
**6**	Rs014500	R1_8001479	pathogenesis-related protein 5-like [BLAST2GO] ǀ Best-Hit: Pathogenesis-related thaumatin superfamily protein [AT1G75050]
**7**	Rs014460	R1_8001479	nucleotide-diphospho-sugar transferase family protein [BLAST2GO] ǀ Nucleotide-diphospho-sugar transferase family protein [AT1G19360]
**8**	Rs014470	R1_8001479	protein brassinazole-resistant 2 [BLAST2GO] ǀ Brassinosteroid signalling positive regulator (BZR1) family protein [AT1G19350]
**9**	Rs014480	R1_8001479	methyltransferase-like protein 2-like [BLAST2GO] ǀ Methyltransferase MT-A70 family protein [AT1G19340]
**10**	Rs014410	R1_8001479	Unknown protein
**11**	Rs014490	R1_8001479	pinus taeda anonymous locus cl4080contig1_02 genomic sequence [BLAST2GO] ǀ Best-Hit: Pathogenesis-related thaumatin superfamily protein [AT1G19320]
**12**	Rs045080	R2_41096883<br>R2_41096959<br>R2_41097093<br>R2_41097103<br>R2_41097111	disease resistance protein adr1-like 1 [BLAST2GO] ǀ Best-Hit: ADR1-like 1 [AT4G33300]
**13**	Rs045160	R2_41096883<br>R2_41096959<br>R2_41097093<br>R2_41097103<br>R2_41097111	zinc ion binding protein [BLAST2GO]
**14**	Rs045070	R2_41096883<br>R2_41096959<br>R2_41097093<br>R2_41097103<br>R2_41097111	glycogenin-like starch initiation protein 3 [BLAST2GO] ǀ plant glycogenin-like starch initiation protein 3 [AT4G33330]
**15**	Rs045110	R2_41096883<br>R2_41096959<br>R2_41097093<br>R2_41097103<br>R2_41097111	eukaryotic translation initiation factor 3 subunit k-like [BLAST2GO] ǀ eukaryotic translation initiation factor 3K [AT4G33250]
**16**	Rs045100	R2_41096883<br>R2_41096959<br>R2_41097093<br>R2_41097103<br>R2_41097111	Best-Hit: F-box family protein [Bra037024]
**17**	Rs045130	R2_41096883<br>R2_41096959<br>R2_41097093	pectinesterase 44 [BLAST2GO] ǀ pectin methylesterase 44 [AT4G33220]

		R2_41097103<br>R2_41097111	
**18**	Rs045120	R2_41096883<br>R2_41096959<br>R2_41097093<br>R2_41097103<br>R2_41097111	1-phosphatidylinositol-3-phosphate 5-kinase-like protein [BLAST2GO] | 1-phosphatidylinositol-3-phosphate 5-kinases [AT4G33240]
**19**	Rs045150	R2_41096883<br>R2_41096959<br>R2_41097093<br>R2_41097103<br>R2_41097111	myosin-h heavy chain-like [BLAST2GO] | myosin putative [AT4G33200]
**20**	Rs045090	R2_41096883<br>R2_41096959<br>R2_41097093<br>R2_41097103<br>R2_41097111	cell division cycle cofactor of apc complex [BLAST2GO] | Transducin family protein / WD-40 repeat family protein [AT4G33270]
**21**	Rs045140	R2_41096883<br>R2_41096959<br>R2_41097093<br>R2_41097103<br>R2_41097111	f-box lrr-repeat protein 15-like [BLAST2GO] | F-box family protein [AT4G33210]
**22**	Rs118270	R3_9521823	vacuolar fusion protein mon1 homolog a-like [BLAST2GO] | SAND family protein [AT2G28390]
**23**	Rs118260	R3_9521823	Unknown protein
**24**	Rs118250	R3_9521823	polyubiquitin [BLAST2GO] | Best-Hit: ubiquitin 11 [AT4G05050]
**25**	Rs118240	R3_9521823	beta-galactosidase 8 [BLAST2GO] | beta-galactosidase 8 [AT2G28470]
**26**	Rs118230	R3_9521823	uncharacterized crm domain-containing protein chloroplastic-like [BLAST2GO] | RNA-binding CRS1 / YhbY (CRM) domain protein [AT2G28480]
**27**	Rs118220	R3_9521823	Best-Hit: Protein of unknown function (DUF295) [AT5G52940]
**28**	Rs160710	R4_6467229	dna-directed rna polymerase ii subunit rpb2-like [BLAST2GO] | DNA-directed RNA polymerase family protein [AT4G21710]
**29**	Rs160760	R4_6467229	subtilase family protein [BLAST2GO] | Best-Hit: Subtilase family protein [AT4G21650]
**30**	Rs173660	R4_14103942	uncharacterized protein [BLAST2GO] | Best-Hit: unknown protein [AT5G38880]
**31**	Rs160700	R4_6467229	defensin-like protein 165 isoform 1 [BLAST2GO] | Best-Hit: unknown protein [AT4G21720]
**32**	Rs173670	R4_14103942	o-fucosyltransferase family protein [BLAST2GO] | O-fucosyltransferase family protein [AT3G30300]
**33**	Rs160730	R4_6467229	nitrate transporter -like [BLAST2GO] | NITRATE TRANSPORTER 1.8 [AT4G21680]
**34**	Rs204540	R4_38574294	low quality protein: heat repeat-containing protein 7a homolog [BLAST2GO] | ARM repeat superfamily protein [AT2G36810]
**35**	Rs205710	R4_38574294	late embryogenesis abundant protein [BLAST2GO] | embryonic cell protein 63 [AT2G36640]
**36**	Rs207870	R4_40395866<br>R4_40395879<br>R4_40395905<br>R4_40395920	transferase family protein [BLAST2GO] | HXXXD-type acyl-transferase family protein [AT2G40230]
**37**	Rs160720	R4_6467229	pentatricopeptide repeat-containing protein [BLAST2GO] | Tetratricopeptide repeat (TPR)-like superfamily protein [AT4G21705]

**38**	Rs173640	R4_14103942	myb domain protein 121 [BLAST2GO] ǀ myb domain protein 121 [AT3G30210]
**39**	Rs204550	R4_38574294	low quality protein: heat repeat-containing protein 7a homolog [BLAST2GO] ǀ ARM repeat superfamily protein [AT2G36810]
**40**	Rs207840	R4_40395866<br>R4_40395879<br>R4_40395905<br>R4_40395920	myb family transcription factor [BLAST2GO] ǀ Homeodomain-like superfamily protein [AT2G40260]
**41**	Rs160750	R4_6467229	splicing factor 3b subunit 2-like [BLAST2GO] ǀ proline-rich spliceosome-associated (PSP) family protein [AT4G21660]
**42**	Rs604630	R4_40395866<br>R4_40395879<br>R4_40395905<br>R4_40395920	kinase superfamily protein isoform 4 [BLAST2GO] ǀ Protein kinase superfamily protein [AT2G40120]
**43**	Rs204520	R4_38574294	glucosyl transferase [BLAST2GO] ǀ Best-Hit: UDP-glucosyl transferase 73C2 [AT2G36760]
**44**	Rs207850	R4_40395866<br>R4_40395879<br>R4_40395905<br>R4_40395920	gdsl esterase lipase at2g30310-like [BLAST2GO] ǀ SGNH hydrolase-type esterase superfamily protein [AT2G40250]
**45**	Rs207910	R4_40395866<br>R4_40395879<br>R4_40395905<br>R4_40395920	probable protein phosphatase 2c 25-like [BLAST2GO] ǀ phosphatase 2C5 [AT2G40180]
**46**	Rs160740	R4_6467229	rna polymerase ii c-terminal domain phosphatase-like 1-like [BLAST2GO] ǀ C-terminal domain phosphatase-like 1 [AT4G21670]
**47**	Rs160690	R4_6467229	uncharacterized protein [BLAST2GO] ǀ Best-Hit: unknown protein [AT4G21740]
**48**	Rs604620	R4_40395866<br>R4_40395879<br>R4_40395905<br>R4_40395920	kinase superfamily protein isoform 4 [BLAST2GO] ǀ Protein kinase superfamily protein [AT2G40120]
**49**	Rs205720	R4_38574294	f-box protein [BLAST2GO] ǀ F-box and associated interaction domains-containing protein [AT3G13680]
**50**	Rs204530	R4_38574294	glucosyl transferase [BLAST2GO] ǀ UDP-glucosyl transferase 73C1 [AT2G36750]
**51**	Rs173650	R4_14103942	protein agamous-like 79 [BLAST2GO] ǀ AGAMOUS-like 79 [AT3G30260]
**52**	Rs207880	R4_40395866<br>R4_40395879<br>R4_40395905<br>R4_40395920	transcription factor bhlh51 [BLAST2GO] ǀ basic helix-loop-helix (bHLH) DNA-binding superfamily protein [AT2G40200]
**53**	Rs204500	R4_38574294	probable 2-oxoglutarate fe -dependent dioxygenase-like [BLAST2GO] ǀ 2-oxoglutarate (2OG) and Fe(II)-dependent oxygenase superfamily protein [AT2G36690]
**54**	Rs207930	R4_40395866<br>R4_40395879<br>R4_40395905<br>R4_40395920	serine threonine protein kinase [BLAST2GO] ǀ Protein kinase superfamily protein [AT2G40120]
**55**	Rs207890	R4_40395866<br>R4_40395879<br>R4_40395905<br>R4_40395920	glycosyl transferase family 1 protein [BLAST2GO] ǀ Best-Hit: phosphatase 2C5 [Bra017025]

**56**	Rs204510	R4_38574294	acyl- n-acyltransferase with ring fyve phd-type zinc finger domain-containing protein [BLAST2GO] ǀ Acyl-CoA N-acyltransferase with RING/FYVE/PHD-type zinc finger domain [AT2G36720]
**57**	Rs160770	R4_6467229	subtilase family protein [BLAST2GO] ǀ Best-Hit: Subtilase family protein [AT4G21650]
**58**	Rs173630	R4_14103942	brassinosteroid-6-oxidase 2 [BLAST2GO] ǀ brassinosteroid-6-oxidase 2 [AT3G30180]
**59**	Rs277810	R5_39670163	proton-dependent oligopeptide transport family protein [BLAST2GO] ǀ Major facilitator superfamily protein [AT3G25260]
**60**	Rs220600	R5_825436 R5_824738	adenine nucleotide alpha hydrolases-like protein [BLAST2GO] ǀ Adenine nucleotide alpha hydrolases-like superfamily protein [AT3G03290]
**61**	Rs220590	R5_825436 R5_824738	endoribonuclease dicer-like 2 [BLAST2GO] ǀ dicer-like 2 [AT3G03300]
**62**	Rs267660	R5_34137688	cell redox homeostasis protein [BLAST2GO] ǀ Thioredoxin protein with domain of unknown function (DUF1692) [AT1G50950]
**63**	Rs267710	R5_34137688	ap2 erf and b3 domain-containing transcription factor [BLAST2GO] ǀ AP2/B3 transcription factor family protein [AT1G51120]
**64**	Rs220690	R5_825436 R5_824738	expansin a13 [BLAST2GO] ǀ expansin A13 [AT3G03220]
**65**	Rs277750	R5_39670163	vacuolar iron transporter homolog 4-like [BLAST2GO] ǀ Vacuolar iron transporter (VIT) family protein [AT3G25190]
**66**	Rs277800	R5_39670163	uncharacterized protein [BLAST2GO] ǀ Protein of unknown function (DUF506) [AT3G25240]
**67**	Rs267670	R5_34137688	Unknown protein
**68**	Rs267700	R5_34137688	transcription factor ilr3 [BLAST2GO] ǀ basic helix-loop-helix (bHLH) DNA-binding superfamily protein [AT1G51070]
**69**	Rs220680	R5_825436 R5_824738	esterase lipase thioesterase family protein [BLAST2GO] ǀ alpha/beta-Hydrolases superfamily protein [AT3G03240]
**70**	Rs220620	R5_825436 R5_824738	uncharacterized protein [BLAST2GO] ǀ Best-Hit: unknown protein [AT3G03280]
**71**	Rs267730	R5_34137688	ap2 erebp transcription factor [BLAST2GO] ǀ Integrase-type DNA-binding superfamily protein [AT1G51190]
**72**	Rs220650	R5_825436 R5_824738	homeobox-leucine zipper protein hdg8 [BLAST2GO] ǀ homeodomain GLABROUS 8 [AT3G03260]
**73**	Rs277780	R5_39670163	helicase-like protein [BLAST2GO]
**74**	Rs223370	R5_2548288	myb domain protein 78 [BLAST2GO] ǀ myb domain protein 108 [AT3G06490]
**75**	Rs267720	R5_34137688	component of smc5 6 dna repair complex [BLAST2GO] ǀ Nse4 component of Smc5/6 DNA repair complex [AT1G51130]
**76**	Rs220640	R5_825436 R5_824738	adenine nucleotide alpha hydrolases-like protein [BLAST2GO] ǀ Adenine nucleotide alpha hydrolases-like superfamily protein [AT3G03270]
**77**	Rs223360	R5_2548288	ribonuclease h protein at1g65750-like [BLAST2GO] ǀ Best-Hit: Ribonuclease H-like superfamily protein [AT4G29090]
**78**	Rs277790	R5_39670163	rotamase fkbp 1 [BLAST2GO] ǀ rotamase FKBP 1 [AT3G25230]
**79**	Rs220700	R5_825436 R5_824738	nac domain containing protein 45 [BLAST2GO] ǀ NAC domain containing protein 45 [AT3G03200]
**80**	Rs267740	R5_34137688	ap2 erebp transcription factor [BLAST2GO] ǀ Integrase-type DNA-binding superfamily protein [AT1G51190]
**81**	Rs223400	R5_2548288	beta-glucosidase-like sfr2 [BLAST2GO] ǀ Glycosyl hydrolase superfamily protein [AT3G06510]
**82**	Rs277760	R5_39670163	peptidyl-prolyl cis-trans isomerase fkbp15-1 [BLAST2GO] ǀ FK506-binding protein 15 kD-1 [AT3G25220]
**83**	Rs277830	R5_39670163	rhodanese cell cycle control phosphatase superfamily protein [BLAST2GO] ǀ Rhodanese/Cell cycle control phosphatase superfamily protein [AT3G25480]

**84**	Rs267680	R5_34137688	gibberellin 2-beta-dioxygenase 8-like [BLAST2GO] ǀ gibberellin 2-oxidase 7 [AT1G50960]
**85**	Rs223410	R5_2548288	uncharacterized protein at3g06530-like [BLAST2GO] ǀ ARM repeat superfamily protein [AT3G06530]
**86**	Rs220670	R5_825436 R5_824738	Unknown protein
**87**	Rs277770	R5_39670163	nucleic acid-ob-fold-like protein [BLAST2GO] ǀ Best-Hit: Nucleic acid-binding OB-fold-like protein [AT1G52950]
**88**	Rs223390	R5_2548288	neutral invertase [BLAST2GO] ǀ Plant neutral invertase family protein [AT3G06500]
**89**	Rs220610	R5_825436 R5_824738	Best-Hit: Adenine nucleotide alpha hydrolases-like superfamily protein [Bra032000]
**90**	Rs277820	R5_39670163	rrna methyltransferase -like [BLAST2GO] ǀ bacterial hemolysin-related [AT3G25470]
**91**	Rs267690	R5_34137688	histone h2a 10 [BLAST2GO] ǀ histone H2A 10 [AT1G51060]
**92**	Rs223420	R5_2548288	rab escort protein [BLAST2GO] ǀ Rab escort protein [AT3G06540]
**93**	Rs220660	R5_825436 R5_824738	udp-glucose pyrophosphorylase [BLAST2GO] ǀ Best-Hit: UDP-GLUCOSE PY-ROPHOSPHORYLASE 1 [AT3G03250]
**94**	Rs357300	R6_47694824 R6_47694893 R6_47694770	uncharacterized protein [BLAST2GO] ǀ Best-Hit: CONTAINS InterPro DO-MAIN/s: Herpesvirus UL139 cytomegalovirus (InterPro: IPR021042) [AT2G34580]
**95**	Rs357220	R6_47694824 R6_47694893 R6_47694770	60s ribosomal protein l18a [BLAST2GO] ǀ Ribosomal protein L18ae/LX family protein [AT2G34480]
**96**	Rs357280	R6_47694824 R6_47694893 R6_47694770	gibberellin 2-oxidase [BLAST2GO] ǀ gibberellin 2-oxidase 3 [AT2G34555]
**97**	Rs357180	R6_47694824 R6_47694893 R6_47694770	pentatricopeptide repeat-containing protein [BLAST2GO] ǀ Pentatricopeptide repeat (PPR-like) superfamily protein [AT2G34400]
**98**	Rs360250	R6_49696836	alpha-galacturonosyltransferase [BLAST2GO] ǀ galacturonosyltransferase 7 [AT2G38650]
**99**	Rs360310	R6_49696836	Best-Hit: unknown protein [AT2G38790]
**100**	Rs357230	R6_47694824 R6_47694893 R6_47694770	cytochrome p450 [BLAST2GO] ǀ cytochrome P450 family 710 subfamily A pol-ypeptide 1 [AT2G34500]
**101**	Rs360260	R6_49696836	ethanolamine-phosphate cytidylyltransferase-like [BLAST2GO] ǀ phosphory-lethanolamine cytidylyltransferase 1 [AT2G38670]
**102**	Rs360300	R6_49696836	intron-binding protein aquarius-like [BLAST2GO] ǀ P-loop containing nucleo-side triphosphate hydrolases superfamily protein [AT2G38770]
**103**	Rs357190	R6_47694824 R6_47694893 R6_47694770	photosystem ii type i chlorophyll a b binding protein [BLAST2GO] ǀ light-har-vesting chlorophyll-protein complex II subunit B1 [AT2G34430]
**104**	Rs357200	R6_47694824 R6_47694893 R6_47694770	photosystem ii type i chlorophyll a b binding protein [BLAST2GO] ǀ light-har-vesting chlorophyll-protein complex II subunit B1 [AT2G34430]
**105**	Rs360270	R6_49696836	Best-Hit: unknown protein [AT2G38690]
**106**	Rs357210	R6_47694824 R6_47694893 R6_47694770	eukaryotic translation initiation factor 1a-like [BLAST2GO] ǀ Best-Hit: Nucleic acid-binding OB-fold-like protein [AT2G04520]
**107**	Rs360280	R6_49696836	peptidyl-prolyl cis-trans isomerase [BLAST2GO] ǀ Cyclophilin-like peptidyl-prolyl cis-trans isomerase family protein [AT2G38730]

**108**	Rs357260	R6_47694824<br>R6_47694893<br>R6_47694770	Best-Hit: unknown protein [AT2G34530]
**109**	Rs360290	R6_49696836	bile acid na+ symporter family protein [BLAST2GO] ǀ Sodium Bile acid symporter family [AT2G26900]
**110**	Rs357270	R6_47694824<br>R6_47694893<br>R6_47694770	binding protein [BLAST2GO] ǀ Best-Hit: unknown protein [AT2G34540]
**111**	Rs357240	R6_47694824<br>R6_47694893<br>R6_47694770	uncharacterized protein [BLAST2GO] ǀ Protein of unknown function DUF642 [AT2G34510]
**112**	Rs360230	R6_49696836	acid phosphatase 1-like [BLAST2GO] ǀ HAD superfamily subfamily IIIB acid phosphatase [AT2G38600]
**113**	Rs360330	R6_49696836	calmodulin-binding protein [BLAST2GO] ǀ Plant calmodulin-binding protein-related [AT2G38800]
**114**	Rs357250	R6_47694824<br>R6_47694893<br>R6_47694770	ribosomal protein s14 [BLAST2GO] ǀ mitochondrial ribosomal protein S14 [AT2G34520]
**115**	Rs357310	R6_47694824<br>R6_47694893<br>R6_47694770	protein tify 5a [BLAST2GO] ǀ jasmonate-zim-domain protein 7 [AT2G34600]
**116**	Rs357290	R6_47694824<br>R6_47694893<br>R6_47694770	nucleoside triphosphatase ccp1 [BLAST2GO] ǀ P-loop containing nucleoside triphosphate hydrolases superfamily protein [AT2G34560]
**117**	Rs360240	R6_49696836	kh domain-containing protein at2g38610-like [BLAST2GO] ǀ RNA-binding KH domain-containing protein [AT2G38610]
**118**	Rs360320	R6_49696836	calmodulin-binding protein [BLAST2GO] ǀ Plant calmodulin-binding protein-related [AT2G38800]
**119**	Rs393670	R7_6987177	monoacylglycerol lipase abhd6-like [BLAST2GO] ǀ Best-Hit: alpha/beta-Hydrolases superfamily protein [AT5G09430]
**120**	Rs393600	R7_6987579<br>R7_6987177	NAC domain containing protein 82 [AT5G09330]
**121**	Rs393500	R7_6987579<br>R7_6987177	ccaat-binding transcription factor (cbf-b nf-ya) family protein [BLAST2GO] ǀ nuclear factor Y subunit A4 [AT2G34720]
**122**	Rs393640	R7_6987579<br>R7_6987177	calmodulin-binding transcription activator 1 [BLAST2GO] ǀ ethylene induced calmodulin binding protein [AT5G09410]
**123**	Rs404760	R7_1433172<br>R7_1433148<br>R7_1433201<br>R7_1434670	homeobox-leucine zipper protein revoluta-like [BLAST2GO] ǀ Homeobox-leucine zipper family protein / lipid-binding START domain-containing protein [AT5G60690]
**124**	Rs393560	R7_6987579<br>R7_6987177	Pectin lyase-like superfamily protein [AT5G09280]
**125**	Rs393570	R7_6987579<br>R7_6987177	gdp-mannose transporter gonst1-like [BLAST2GO] ǀ Best-Hit: CONTAINS InterPro DOMAIN/s: Gamma-secretase aspartyl protease complex presenilin enhancer-2 subunit (InterPro: IPR019379) [AT5G09310]
**126**	Rs393490	R7_6987579<br>R7_6987177	ataf2 protein [BLAST2GO] ǀ NAC (No Apical Meristem) domain transcriptional regulator superfamily protein [AT5G08790]
**127**	Rs404750	R7_1433172<br>R7_1433148<br>R7_1433201<br>R7_1434670	uncharacterized protein [BLAST2GO] ǀ Protein of unknown function DUF584 [AT5G60680]
**128**	Rs384710	R7_11871666	subtilase family protein [BLAST2GO] ǀ Subtilase family protein [AT1G66220]

**129**	Rs393580	R7_6987579<br>R7_6987177	pentatricopeptide repeat-containing protein at3g46870-like [BLAST2GO] | Vacuolar sorting protein 9 (VPS9) domain [AT5G09320]
**130**	Rs393530	R7_6987579<br>R7_6987177	rna polymerase ii transcriptional coactivator kiwi [BLAST2GO] | ssDNA-binding transcriptional regulator [AT5G09250]
**131**	Rs393540	R7_6987579<br>R7_6987177	snf7 family protein [BLAST2GO] | vacuolar protein sorting-associated protein 20.2 [AT5G09260]
**132**	Rs393520	R7_6987579<br>R7_6987177	amino acid transporter aap4 [BLAST2GO] | amino acid permease 2 [AT5G09220]
**133**	Rs393620	R7_6987579<br>R7_6987177	dna-directed rna polymerase iii rpc4 family protein [BLAST2GO] | RNA polymerase III RPC4 [AT5G09380]
**134**	Rs393630	R7_6987579<br>R7_6987177	cd2 antigen cytoplasmic tail-binding protein 2 [BLAST2GO] | CD2-binding protein-related [AT5G09390]
**135**	Rs393510	R7_6987579<br>R7_6987177	Best-Hit: Nucleic acid-binding OB-fold-like protein [Bra036277]
**136**	Rs384720	R7_11871666	subtilase family protein [BLAST2GO] | Subtilisin-like serine endopeptidase family protein [AT1G66210]
**137**	Rs393550	R7_6987579<br>R7_6987177	uncharacterized protein [BLAST2GO] | Best-Hit: unknown protein [AT5G09270]
**138**	Rs393590	R7_6987579<br>R7_6987177	guanine nucleotide exchange factor vps9a [BLAST2GO] | Best-Hit: Vacuolar sorting protein 9 (VPS9) domain [AT3G19770]
**139**	Rs393660	R7_6987579<br>R7_6987177	outer envelope protein mitochondrial-like [BLAST2GO] | translocon at the outer membrane of chloroplasts 64-V [AT5G09420]
**140**	Rs404770	R7_1433172<br>R7_1433148<br>R7_1433201<br>R7_1434670	glycosyltransferase family protein 2 [BLAST2GO] | glycosyltransferase family protein 2 [AT5G60700]
**141**	Rs393610	R7_6987579<br>R7_6987177	laccase family protein diphenol oxidase family protein [BLAST2GO] | Best-Hit: laccase 14 [AT5G09360]
**142**	Rs410230	R8_2129514	Unknown protein
**143**	Rs410270	R8_2129514	starch synthase 3 [BLAST2GO] | starch synthase 3 [AT1G11720]
**144**	Rs410240	R8_2129514	Unknown protein
**145**	Rs410250	R8_2129514	sterol 14-demethylase [BLAST2GO] | CYTOCHROME P450 51G1 [AT1G11680]
**146**	Rs410260	R8_2129514	uncharacterized protein [BLAST2GO] | Best-Hit: unknown protein [AT1G11690]
**147**	Rs410310	R8_2129514	arogenate dehydratase prephenate dehydratase chloroplastic-like [BLAST2GO] | arogenate dehydratase 1 [AT1G11790]
**148**	Rs410300	R8_2129514	mediator of rna polymerase ii transcription subunit 32-like [BLAST2GO] | Best-Hit: MED32 [AT1G11760]
**149**	Rs410330	R8_2129514	glyoxalase i homolog [BLAST2GO] | glyoxalase I homolog [AT1G11840]
**150**	Rs410280	R8_2129514	ankyrin repeat family protein [AT1G11740]
**151**	Rs410320	R8_2129514	glucan endo- -beta-glucosidase 1-like [BLAST2GO] | O-Glycosyl hydrolases family 17 protein [AT1G11820]
**152**	Rs410290	R8_2129514	atp-dependent clp protease proteolytic subunit 6 [BLAST2GO] | CLP protease proteolytic subunit 6 [AT1G11750]
**153**	Rs454250	R9_3971141	xylosyltransferase 1-like [BLAST2GO] | Core-2/I-branching beta-1 6-N-acetylglucosaminyltransferase family protein [AT1G71070]
**154**	Rs454010	R9_3971141	multi-copper oxidase type i family protein [BLAST2GO] | Cupredoxin superfamily protein [AT1G71040]
**155**	Rs448400	R9_204910	uncharacterized protein [BLAST2GO] | Best-Hit: unknown protein [AT1G80610]
**156**	Rs448420	R9_204910	3-isopropylmalate dehydrogenase [BLAST2GO] | isopropylmalate dehydrogenase 2 [AT1G80560]

**157**	Rs454220	R9_3971141	uncharacterized protein [BLAST2GO] ǀ Best-Hit: unknown protein [AT1G71110]
**158**	Rs454020	R9_3971141	spore coat protein [BLAST2GO] ǀ Cupredoxin superfamily protein [AT1G71040]
**159**	Rs498780	R9_36512482	tir-nbs-lrr class disease resistance protein [BLAST2GO] ǀ Disease resistance protein (TIR-NBS-LRR class) family [AT4G11170]
**160**	Rs498770	R9_36512482	arf gtpase-activating domain-containing protein [BLAST2GO] ǀ ARF-GAP domain 2 [AT1G60860]
**161**	Rs448410	R9_204910	acetylornithine mitochondrial-like [BLAST2GO] ǀ HOPW1-1-interacting 1 [AT1G80600]
**162**	Rs454030	R9_3971141	multi-copper oxidase type i family protein [BLAST2GO] ǀ Cupredoxin superfamily protein [AT1G71040]
**163**	Rs448370	R9_204910	probable receptor-like protein kinase at1g80640-like [BLAST2GO] ǀ Protein kinase superfamily protein [AT1G80640]
**164**	Rs449680	R9_204910	Best-Hit: unknown protein [AT1G67020]
**165**	Rs498740	R9_36512482	dna-directed rna polymerase subunit aac42 [BLAST2GO] ǀ DNA-directed RNA polymerase family protein [AT1G60850]
**166**	Rs454050	R9_3971141	myb-like transcription factor 2 [BLAST2GO] ǀ MYB-like 2 [AT1G71030]
**167**	Rs448460	R9_204910	f-box kelch-repeat protein [BLAST2GO] ǀ Best-Hit: F-box family protein [AT1G25150]
**168**	Rs454210	R9_3971141	cyclin-d1-binding protein 1 homolog [BLAST2GO] ǀ Best-Hit: unknown protein [AT1G71150]
**169**	Rs454260	R9_3971141	heavy-metal-associated domain-containing protein [BLAST2GO] ǀ Heavy metal transport/detoxification superfamily protein [AT1G71050]
**170**	Rs448380	R9_204910	rni-like superfamily protein [BLAST2GO] ǀ Best-Hit: RNI-like superfamily protein [AT1G80630]
**171**	Rs454200	R9_3971141	beta-ketoacyl- synthase family protein [BLAST2GO]
**172**	Rs454230	R9_3971141	auxin efflux carrier family protein [BLAST2GO] ǀ Auxin efflux carrier family protein [AT1G71090]
**173**	Rs498800	R9_36512482	phosphatidylinositol-4-phosphate 5-kinase family protein [BLAST2GO] ǀ Phosphatidylinositol-4-phosphate 5-kinase family protein [AT1G60890]
**174**	Rs448450	R9_204910	major facilitator superfamily protein [BLAST2GO] ǀ Major facilitator superfamily protein [AT1G80530]
**175**	Rs448390	R9_204910	s15 rna-binding protein [BLAST2GO] ǀ S15/NS1 RNA-binding protein [AT1G80620]
**176**	Rs448470	R9_204910	sterile alpha motif domain-containing protein [BLAST2GO] ǀ Sterile alpha motif (SAM) domain-containing protein [AT1G80520]
**177**	Rs454240	R9_3971141	rna polymerase ii transcription elongation factor [BLAST2GO] ǀ RNA polymerase II transcription elongation factor [AT1G71080]
**178**	Rs448440	R9_204910	uncharacterized protein [BLAST2GO] ǀ Best-Hit: unknown protein [AT1G80540]
**179**	Rs448360	R9_204910	h(+)-atpase 9 [BLAST2GO] ǀ H(+)-ATPase 9 [AT1G80660]
**180**	Rs498750	R9_36512482	Unknown protein
**181**	Rs448430	R9_204910	pentatricopeptide repeat-containing protein [BLAST2GO] ǀ Pentatricopeptide repeat (PPR) superfamily protein [AT1G80550]

Table S4. Disease-related genes identified 225 Kb upstream and downstream of the SNP loci associated with Fusarium wilt of radish.

No.	Genes	SNP	Function
**1**	Rs014540	R1_8001479	zinc finger family protein [BLAST2GO] ǀ RING/U-box super-family protein [AT1G19310]
**2**	Rs044300	R2_41096883 R2_41096959 R2_41097093 R2_41097103 R2_41097111	zinc finger swim domain-containing protein 7-like [BLAST2GO] ǀ Best-Hit: unknown protein [AT4G33925]
**3**	Rs044340	R2_41096883 R2_41096959 R2_41097093 R2_41097103 R2_41097111	leucine-rich repeat family protein [BLAST2GO] ǀ Leucine-rich repeat (LRR) family protein [AT4G33970]
**4**	Rs045140	R2_41096883 R2_41096959 R2_41097093 R2_41097103 R2_41097111	f-box lrr-repeat protein 15-like [BLAST2GO] ǀ F-box family protein [AT4G33210]
**5**	Rs160400	R4_6467229	tir-nbs-lrr class disease resistance protein [BLAST2GO] ǀ Best-Hit: Disease resistance protein (TIR-NBS-LRR class) [AT5G11250]
**6**	Rs160410	R4_6467229	disease resistance protein [BLAST2GO] ǀ Best-Hit: Disease resistance protein (TIR-NBS-LRR class) [AT5G11250]
**7**	Rs160430	R4_6467229	tir-nbs-lrr class disease resistance protein [BLAST2GO] ǀ Best-Hit: disease resistance protein (TIR-NBS-LRR class) [AT1G69550]
**8**	Rs160440	R4_6467229	disease resistance protein [BLAST2GO] ǀ Best-Hit: Disease resistance protein (TIR-NBS-LRR class) [AT5G11250]
**9**	Rs160600	R4_6467229	tir-nbs-lrr class disease resistance protein [BLAST2GO] ǀ Best-Hit: disease resistance protein (TIR-NBS-LRR class) [AT1G69550]
**10**	Rs160610	R4_6467229	disease resistance protein [BLAST2GO] ǀ Best-Hit: Disease resistance protein (TIR-NBS-LRR class) [AT5G11250]
**11**	Rs174020	R4_14103942	zinc finger (C2H2 type) family protein [AT3G29340]
**12**	Rs204510	R4_38574294	acyl- n-acyltransferase with ring fyve phd-type zinc finger domain-containing protein [BLAST2GO] ǀ Acyl-CoA N-acyltransferase with RING/FYVE/PHD-type zinc finger domain [AT2G36720]
**13**	Rs205540	R4_38574294	protein [BLAST2GO] ǀ Best-Hit: Disease resistance protein (TIR-NBS-LRR class) family [AT3G44480]
**14**	Rs208240	R4_40395866 R4_40395879 R4_40395905 R4_40395920	zinc finger protein 8 [BLAST2GO] ǀ zinc finger protein 8 [AT2G41940]
**15**	Rs208270	R4_40395866 R4_40395879 R4_40395905 R4_40395920	nucleic acid binding [BLAST2GO] ǀ CCCH-type zinc finger protein with ARM repeat domain [AT2G41900]
**16**	Rs208380	R4_40395866 R4_40395879 R4_40395905 R4_40395920	zinc finger an1 and c2h2 domain-containing stress-associated protein 16-like [BLAST2GO] ǀ zinc finger (C2H2 type AN1-like) family protein [AT2G41835]
**17**	Rs220320	R5_825436 R5_824738	zinc finger family protein [BLAST2GO] ǀ Best-Hit: zinc finger (C2H2 type) family protein [AT3G02790]
**18**	Rs220370	R5_825436 R5_824738	zinc finger protein [BLAST2GO] ǀ Best-Hit: zinc ion binding [AT3G02860]
**19**	Rs223270	R5_2548288	zinc finger ccch domain-containing protein 34 [BLAST2GO] ǀ Zinc finger C-x8-C-x5-C-x3-H type family protein [AT3G06410]
**20**	Rs223290	R5_2548288	zinc finger ccch domain-containing protein 34 [BLAST2GO] ǀ Zinc finger C-x8-C-x5-C-x3-H type family protein [AT3G06410]

**21**	Rs223530	R5_2548288	e3 ubiquitin-protein ligase march6-like [BLAST2GO] ǀ RING/FYVE/PHD zinc finger superfamily protein [AT4G32670]
**22**	Rs223540	R5_2548288	RING/FYVE/PHD zinc finger superfamily protein [AT4G32670]
**23**	Rs267470	R5_34137688	ring fyve phd zinc finger-containing protein [BLAST2GO] ǀ RING/FYVE/PHD zinc finger superfamily protein [AT1G50440]
**24**	Rs267560	R5_34137688	phd finger family protein [BLAST2GO] ǀ RING/FYVE/PHD zinc finger superfamily protein [AT1G50620]
**25**	Rs277930	R5_39670163	zinc finger family protein [BLAST2GO] ǀ DHHC-type zinc finger family protein [AT3G48760]
**26**	Rs357040	R6_47694824 R6_47694893 R6_47694770	ring fyve phd zinc finger superfamily protein [BLAST2GO] ǀ RING/FYVE/PHD zinc finger superfamily protein [AT2G34200]
**27**	Rs357080	R6_47694824 R6_47694893 R6_47694770	ring fyve phd zinc finger superfamily protein [BLAST2GO] ǀ RING/FYVE/PHD zinc finger superfamily protein [AT2G34200]
**28**	Rs360370	R6_49696836	probable lysine-specific demethylase jmj14-like [BLAST2GO] ǀ Transcription factor jumonji (jmj) family protein / zinc finger (C5HC2 type) family protein [AT2G38950]
**29**	Rs360390	R6_49696836	c3hc4-type ring finger-containing protein [BLAST2GO] ǀ Best-Hit: Zinc finger (C3HC4-type RING finger) family protein [AT2G38970]
**30**	Rs360400	R6_49696836	c3hc4-type ring finger-containing protein [BLAST2GO] ǀ Best-Hit: Zinc finger (C3HC4-type RING finger) family protein [AT2G38970]
**31**	Rs384690	R7_11871666	zinc finger protein 4 [BLAST2GO] ǀ zinc finger protein 4 [AT1G66140]
**32**	Rs393460	R7_6987579 R7_6987177	ring fyve phd zinc finger superfamily protein [BLAST2GO] ǀ RING/FYVE/PHD zinc finger superfamily protein [AT5G08750]
**33**	Rs404260	R7_1433172 R7_1433148 R7_1433201 R7_1434670	protein sensitive to proton rhizotoxicity 1-like [BLAST2GO] ǀ C2H2 and C2HC zinc fingers superfamily protein [AT5G22890]
**34**	Rs404330	R7_1433172 R7_1433148 R7_1433201 R7_1434670	disease resistance protein rpp8 [BLAST2GO] ǀ Best-Hit: Disease resistance protein (CC-NBS-LRR class) family [AT5G35450]
**35**	Rs404380	R7_1433172 R7_1433148 R7_1433201 R7_1434670	f-box fbd lrr-repeat protein [BLAST2GO] ǀ Best-Hit: FBD F-box Skp2-like and Leucine Rich Repeat domains containing protein [AT5G22660]
**36**	Rs404440	R7_1433172 R7_1433148 R7_1433201 R7_1434670	c2h2-like zinc finger protein [BLAST2GO] ǀ C2H2-like zinc finger protein [AT5G22990]
**37**	Rs404490	R7_1433172 R7_1433148 R7_1433201 R7_1434670	e3 sumo-protein ligase siz1 [BLAST2GO] ǀ DNA-binding protein with MIZ/SP-RING zinc finger PHD-finger and SAP domain [AT5G60410]
**38**	Rs404510	R7_1433172 R7_1433148 R7_1433201 R7_1434670	e3 sumo-protein ligase siz1 [BLAST2GO] ǀ DNA-binding protein with MIZ/SP-RING zinc finger PHD-finger and SAP domain [AT5G60410]
**39**	Rs498780	R9_36512482	tir-nbs-lrr class disease resistance protein [BLAST2GO] ǀ Disease resistance protein (TIR-NBS-LRR class) family [AT4G11170]
**40**	Rs613740	R7_11871666	zinc finger protein 4 [BLAST2GO] ǀ zinc finger protein 4 [AT1G66140]
